# The effects of medicinal and food homologous substances on blood lipid and blood glucose levels and liver function in patients with nonalcoholic fatty liver disease: a systematic review of randomized controlled trials

**DOI:** 10.1186/s12944-023-01900-5

**Published:** 2023-08-29

**Authors:** Qian Zhang, Yatian Jia, Yuexing Zhang, Yan Wang, Xinru Li, Xiaoying Tian, Shifan Han

**Affiliations:** 1 General Surgery Department, Shanxi Bethune Hospital/Third Hospital of Shanxi Medical University, Taiyuan, China; 2 School of Nursing/Research Center of Dietary Therapy Technology, Shanxi Medical University, Taiyuan, China; 3 School of Nursing, Shanxi University of Chinese Medicine, Jinzhong, China; 4 School of Nursing, Peking University, Beijing, China; 5 Department of Infection and Toxic Liver Diseases, The Third People’s Hospital of Taiyuan, Taiyuan, China; 6 First Hospital of Shanxi Medical University, Taiyuan, China

**Keywords:** Medicinal and food homologous substance, Nonalcoholic fatty liver disease, Blood lipids, Blood glucose, Liver function

## Abstract

**Background:**

Nonalcoholic fatty liver disease (NAFLD) is a prevalent chronic liver disorder worldwide. According to several previous studies, the treatment of patients with NAFLD using medicinal and food-homologous substances has consistent effects on the levels of blood lipids and blood glucose and liver function.

**Objective:**

This systematic review was conducted to investigate the impact of medicinal and food homologous substances on blood lipid and glucose levels as well as liver function in patients with NAFLD.

**Methods:**

A thorough search was conducted in eight databases, including China Science and Technology Journal Database (VIP), Chinese National Knowledge Infrastructure(CNKI), China Biomedical Literature Database (SinoMed), Wanfang Database, PubMed, Cochrane Library, Web of Science and Embase, for articles published from database inception until June 24, 2023. The methodological quality of the included studies was evaluated utilizing Cochrane Randomized Trial Risk Bias Tool, Edition 2 and GRADE methodology for assessment.

**Results:**

A total of 13 randomized controlled trials, involving 829 patients with NAFLD, were included in the analysis, these studies included a total of 9 medicinal and food homologous substances. In the 13 studies, hawthorn (2), sea buckthorn (1), ginger (2), turmeric (4) (1 with chicory seeds), cinnamon (1), cardamom (1), purslane (1) and saffron (1) were included. The results of the included studies showed that medicinal and food homologous substances could improve high-density lipoprotein cholesterol (HDL-C), low-density lipoprotein cholesterol (LDL-C), total cholesterol (TC), triglycerides (TGs), fasting blood glucose (FBG) and liver enzyme levels in patients with NAFLD to a certain extent, but the effect of turmeric on TC, liver enzyme levels is controversial.

**Conclusion:**

In patients with NAFLD, dietary intervention using medicinal and food homologous substances can ameliorate blood lipid and blood glucose levels and liver enzymes to some extent. In clinical work, medicinal and food homologous substances can be used to provide patients with NAFLD with a safe and effective dietary plan to help prevent and treat disease onset and progression.

**Supplementary Information:**

The online version contains supplementary material available at 10.1186/s12944-023-01900-5.

## Background

Fatty liver and hepatic steatosis are two cardinal features of NAFLD. Symptoms of NAFLD encompass anorexia, fatigue, dull abdominal pain, and satiety. The condition may progress to fibrosis, cirrhosis, and hepatocellular carcinoma, thus representing a significant etiology of end-stage liver disease that necessitates liver transplantation with considerable morbidity [1]. With changes in lifestyle and dietary patterns, the incidence of NAFLD is increasing annually and exhibiting a trend towards younger age groups. Currently, NAFLD has become an important public health problem affecting human health, and its disease and economic burden may increase in the next few decades [2]. In 2020, a global expert council suggested renaming NAFLD metabolic-associated fatty liver disease (MAFLD). Although the etiology of NAFLD remains elusive, the disease has been linked to a number of comorbidities, including metabolic syndrome, obesity, cardiovascular disease and diabetes [3, 4]. The development of NAFLD is significantly influenced by metabolic diseases, such as hypertension, dyslipidemia, and abnormal blood glucose levels [5]. To address this significant growth of NAFLD as a major medical concern, multidisciplinary research has shifted its focus to finding effective prevention and treatment methods. During NAFLD treatment, the main focus is on protecting the liver, lowering enzymes, regulating lipids, and lowering blood glucose while coordinating lifestyle, diet adjustments, and exercise. These measures are essential in the management of NAFLD, either for its control or to prevent its progression towards cirrhosis and hepatocellular carcinoma. However, the overall effectiveness remains poor due to the lack of targeted medications and patients' reluctance to modify their diet and lifestyle [6, 7]. In view of the increasing prevalence of NAFLD, it is imperative to discover safe, efficacious, affordable and simple methods for treating and slowing its progression.

Medicine and food with the same origin are defined as homologous. Furthermore, medicinal homologous food can be consumed as food or medicine to treat diseases [8]. When used to treat diseases, medicinal homologous foods have almost no hazardous side effects because they are mostly consumed as food in daily life [9]. The efficacy of these medicines is categorized into three types: upper, middle, and lower grades in Shennong's Materia Medica, which was written by numerous medical scientists in ancient China. Many drugs from the upper grade are still used frequently. These substances, including cassia bark (Chinese cinnamon), ginger, licorice, and wolfberry, are listed in the "Catalogue of Homologous Substances in Food and Traditional Chinese Medicine" issued by the National Health Commission [10, 11], and these medicinal and food homologous substances have certain effects on disease prevention and control [12, 13]. Based on the principle of food and medicine sharing the same origin in traditional Chinese medicine, this study proposes the hypothesis that medicinal and food homologous substances can improve the levels of blood lipids, blood glucose and liver function in patients with NAFLD and discusses their influence on the disease, aiming at providing safe and effective dietary treatment programs for patients with NAFLD to assist in the prevention and treatment of the disease.

## Methods

This study was registered with PROSPERO (Registration Number: CRD42023384871) (https://www.crd.york.ac.uk/PROSPERO/#loginpage) and prepared following the Preferred Reporting Items for Systematic Reviews and Meta-Analyses (PRISMA) guidelines [14].

### Inclusion criteria

Participants (P): adults over 18 years who have undergone imaging or pathological examinations and have been diagnosed with NAFLD.

Intervention (I): The intervention group received dietary therapy intervention using substances in the 17th edition of the Homology Catalog of drug-containing foods [15] and the Notice on Piloting the substance Management of 9 substances, including Codonium codontiana [16], issued by China's National Health Commission and State Administration for Market Regulation on January 2, 2020 (It can be formulated into capsules through the process of grinding). It contains 95 medicinal and food homologous substances.

Control (C): The control group received conventional treatment or placebo.

Outcome indicators (O): (1)Blood lipids: serum HDL-C, LDL-C, TC, and TGs levels; (2)FBG levels; (3)Liver function: aspartate aminotransferase (AST) and alanine aminotransferase (ALT) levels.

Type of studies(S): prospective randomized controlled trials (RCTs).

Exclusion criteria (1) intervention substances are extracts of medicinal and food homologous substances; (2) studies for which data extraction was not possible; (3) repeat published research; and (4) reviews, notices, and conference papers.

### Search strategies

In this study, two researchers (Yatian Jia and Yuexing Zhang) conducted a comprehensive literature search across multiple databases including CNKI, VIP, CBM, Wanfang, PubMed, The Cochrane Libraries, Web of Science and Embase by utilizing MeSH term "non-alcoholic fatty liver disease" along with its corresponding free words and the names of medicinal and food homologous substances. The search period covered from the inception of each database up until June 24, 2023. The search strategy is shown in the additional file 1.

### Literature screening and data extraction

Two researchers (Yatian Jia and Yuexing Zhang) conducted independent literature searches and utilized EndNote20 literature management software to eliminate any duplicate data. Relevant articles were selected based on the analysis of their title and abstract, while irrelevant ones were excluded. The final literature for the analysis was shortlisted after reading the full texts and applying inclusion and exclusion criteria. Two researchers independently retrieved information from the literature, such as the name of first author, country and year of publication, research subjects, sample size, intervention, duration of the intervention, and outcome indicators. Any discrepancies during the screening and data extraction were resolved by consultation with a third researcher.

### Risk of bias assessment

Two researchers (Qian Zhang and Yatian Jia) evaluated the Cochrane Randomized Trial Risk Bias Tool, Edition 2 (RoB 2), and the included literature was evaluated using evaluation criteria. Evaluation included the randomization process, deviation from expected interventions, missing outcome data, outcome measurement, and selection of reported outcomes [17]. Based on the results of RoB 2, the included articles were categorized as "high risk," "some concerns," or "low risk." In case of disagreement in the above process, the third researcher arbitrated, and a consensus was finally reached.

### Evidence quality assessment

Two researchers (Qian Zhang and Yatian Jia) utilized the GRADE (Grading of Recommendations, Assessment, Development and Evaluations) approach [17] to ascertain the certainty of the body of evidence. In this methodology, the initial rating for the quality of evidence from RCTs is high, but it may be downgraded to medium, low or very low if any limitations are identified in terms of bias, inconsistency, directness, imprecision and other aspects. However, evidence can be upgraded with large effects and clear dose–response relationships. If there is disagreement during this process, a third researcher will arbitrate until consensus is reached.

## Results

### Literature search results

**Fig. 1:**
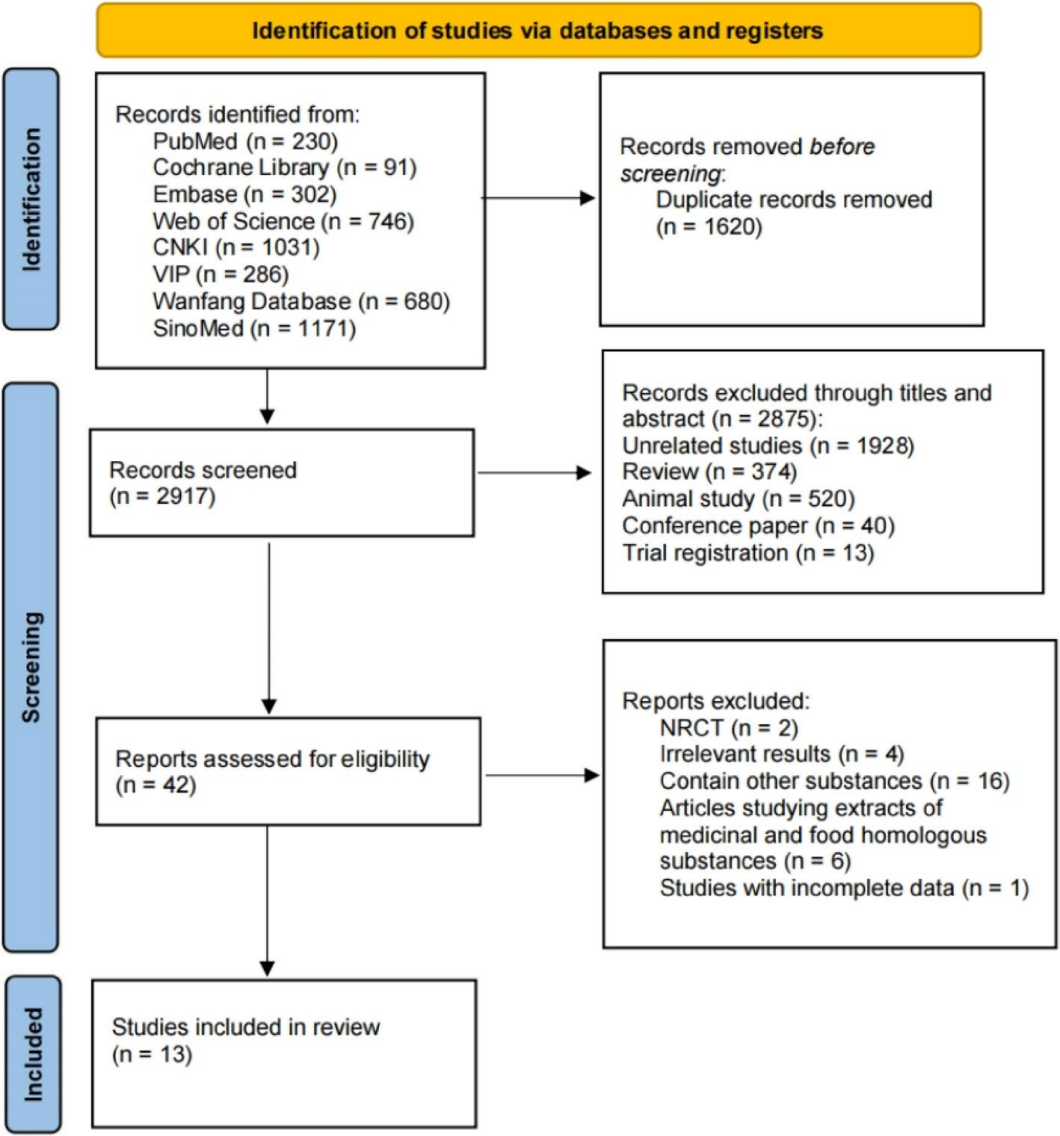
Flow chart of the screening and selection of literature

An initial database search revealed 4,537 relevant publications (3,168 in Chinese and 1,369 in English). After eliminating 1,620 duplicate publications, 2,917 publications were retrieved. After reading the titles and abstracts, 2,875 publications were excluded due to the nonfulfillment of inclusion requirements. A total of 42 research articles fulfilled the inclusion criteria. After a second reading of the entire manuscript, 29 were excluded, and finally 13 articles were included in the study. Among the 13 articles, three [18–20] and 10 [21–30] were in Chinese and English, respectively. Figure 1 shows the literature screening procedure and results.

### Basic characteristics of the included literature

This study included a total of 829 individuals in 13 RCTs (Table 1). The medicinal and food homologous substances included hawthorn (*n* = 2), sea buckthorn (*n* = 1), ginger (*n* = 2), cardamom (*n* = 1), turmeric (*n* = 4), cinnamon (*n* = 1), purslane (*n* = 1), and saffron (*n* = 1). In one of the studies [26], three experimental groups were intervened separately using chicory, turmeric, and chicory + turmeric.

**Table 1:** Basic characteristics of the included literature

First Author, Publication Year, Country	Study Design	Sex (male/ female) (T/C)	Patients	Intervention	Control Condition	Outcomes	Time points of Measurements
Liu Mengyue [18] 2022 China	RCT	T:12/18 C:10/20	98 adult patients with MAFLD, medical examination center I: 50 patients C: 48 patients Age: I: 44.44 ± 12.08 C: 40.84 ± 9.30	Intervention: Tartary buckwheat + sea buckthorn vinegar drink Provider: Researcher Time: 3 months	Monthly group online health education	a, b, c, d, e, f, g	Before/after 3 months
Liu Luming [19] 2012 China	RCT	T:34/16 C:27/21	87 adult patients with NAFLD, College physical examination center I: 45 patients C: 42 patients Age: Unreported	Intervention: The experimental group drank 1 serving of hawthorn tea every day and practiced 36 type tai Chi soft ball for 40 min every day Provider: Researcher Time: 90 days	Type 36 Tai Chi soft force ball	a, b, c, d, e, f, g	Before/after 3 months
Tao Zhenghui [20] 2021 China	RCT	Unreported	60 adult patients with NAFLD, Hospital I: 30 patients C: 30 patients Age: I: 41.00 ± 2.33 C: 40.00 ± 2.30	Hawthorn, 60 g, in 2000 ml of water; boil and drink tea daily Provider: Researcher Time: 3 months	Conventional therapy	a, b, c, d	Before/after 3 months
Roya Rafie [21] 2020 Iron	RCT	Unreported	46 adult patients, Outpatient Department of the gastroenterology clinic I: 23 C: 23 Age: I: 50.04 ± 10.26 C: 47.95 ± 9.24	Capsules of ginger (1500 mg/day) Provider: Researcher Time: 12 weeks	capsules of placebo (1500 mg/day)	a, b, c, d, e, f, g	Before/after 12 weeks
Milad Daneshi-Maskooni [22] 2019 Iron	RCT	T:27/16 C:27/17	87 adult patients were enrolled general clinic I: 43 C: 44 Age: 30–60 years,	Two cardamom capsules 500 mg 3 times daily Provider: Researcher Time: 3 months	Identical placebos (starch)3 g	a, b, c, d, e	Before/after 3 months
Maryam jarhahzadeh [23] 2021 Iron	RCT	T:19/13 C:19/13	64 adult patients were enrolled at a hospital I: 32 C: 32 Age: I: 44.12 ± 8.35 C: 38.56 ± 10.43	Supplemented with Turmeric (2 g/daily) Provider: Researcher Time: 8 weeks	Identical placebos (starch)	a, b, c, d, e	Before/after 8 weeks
Mehran Rahimlou [24] 2016 Iron	RCT	T:11/11 C:9/13	44 adult patients were enrolled at a hospital I: 23 C: 21 Age: I: 45.45 ± 2.31 C: 45 ± 2.14	Capsules of ginger (2000 mg/day) Provider: Researcher Time: 12 weeks	Identical placebos (starch)	f, g	Before/after 12 weeks
Aida Ghaffari [25] 2019 Iron	RCT	T1:10/11 T2:9/12 T3:14/8 C:13/8	44 adult patients were enrolled at a hospital TUR: CHI: TUR + CHI: PLA = 21: 21: 22: 21 Age: TUR: 42.5 ± 6.93 CHI: 41.0 ± 8.61 TUR + CHI: 41.5 ± 7.68 PLA group: 40.3 ± 9.26	TUR: 3 g/d turmeric; CHI: 9 g/d of powdered chicory seed; TUR + CHI: turmeric and chicory seed Provider: Researcher Time: 12 weeks	Identical placebos (starch)	a, b, c, d, f, g	Before/after 12 weeks
Aida Ghaffari [26] 2017 Iron	RCT	T:11/10 C:8/13	44 adult patients were enrolled at a hospital I: 21 C: 21 Age: I: 42.09 ± 7.23 C: 40.38 ± 9.26	3 g of turmeric Provider: Researcher Time: 12wekks	Placebo	e	Before/after 12 weeks
Roya Navekar [27] 2017 Iron	RCT	T:10/11 C:8/13	44 adult patients were enrolled at a clinic I: 21 C: 21 Age: T: 42.09 ± 7.23 C: 40.38 ± 9.26	Six turmeric capsules daily Provider: ResearcherTime: 12 weeks	Placebo	e, f, g	Before/after 12 weeks
Faezeh Askari [28] 2013 Iron	RCT	T:11/12 C:11/11	45 adult patients were enrolled at a clinic I: 23 C: 22 Age: T: 44.8 ± 8.5 C: 45.4 ± 8.2	Capsules of cinnamon (1500 mg/day) Provider: ResearcherTime: 8 weeks	Two placebo capsules (wheat flour)	a, b, c, d, e, f, g	Before/after 12 weeks
Alireza Gheflati [29] 2019 Iron	RCT	Unreported	54 adult patients were enrolled at a clinic I: 27 C: 27 Age: I: 40.07 ± 9.52 C: 39.81 ± 8.84	20 g/day of purslane seeds + low‐ calorie diet Provider: ResearcherTime: 8 weeks	The low‐calorie diet	a, b, c, d, e	2W/4W/8W
Farnaz Kaviani Pour [30] 2020 Iron	RCT	T:21/17 C:22/16	72 adult patients were enrolled at a clinic I: 36 C: 36 Age: I: 43.42 ± 10.62 C: 42.05 ± 8.27	100 mg saffron Provider: ResearcherTime: 12 weeks	Placebo	f, g	Before/after 12 weeks

*NAFLD* Nonalcoholic fatty liver disease, *MAFLD*, Metabolic-associated fatty liver disease, *TC* Total cholesterol, *TGs* Triglycerides, *LDL-C* Low-density lipoprotein cholesterol, *HDL-C* High-density lipoprotein cholesterol, *FBG* Fasting blood glucose, *AST* Aspartate aminotransferase, *ALT* Alanine aminotransferase, *PLA* Placebo group a.*TC* b. *TGs* c. *HDL-C* d. *LDL-C* e. *FBG* f. *AST* g. *ALT*

### Quality assessment of studies

**Fig. 2:**
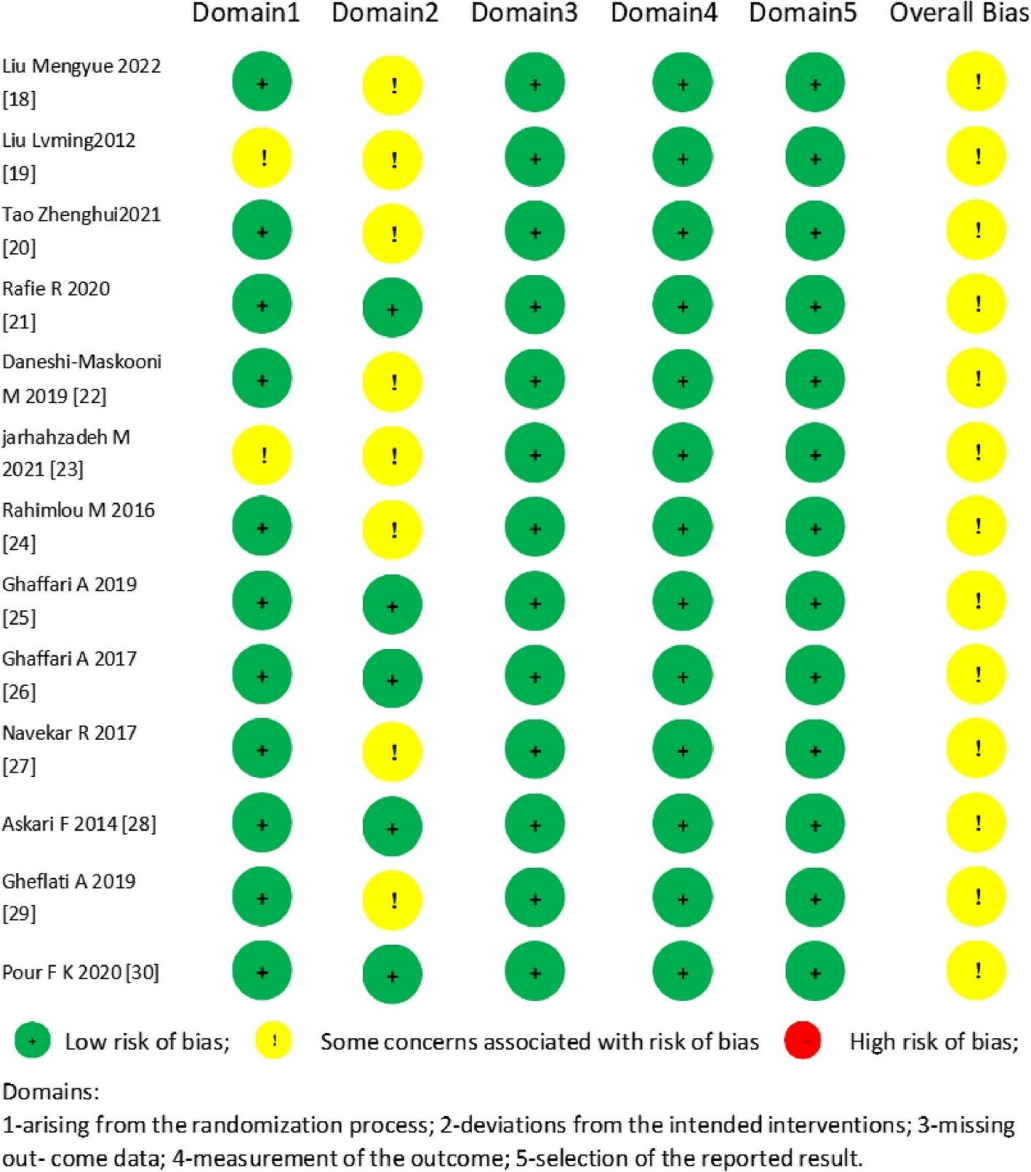
Assessment of risk of bias

Eleven studies [18, 20–22, 24–30] introduced the random allocation technique. Allocation concealment was implemented in six of the included studies [21, 25, 26, 28–30]. In seven investigations [21, 24–28, 30], participants, researchers, and outcome assessors were blinded, whereas in two studies [22, 23], only participants and researchers were blinded. Furthermore, all 13 studies [18–30] explained reasons for lost follow-ups or dropouts. The results of the methodological quality assessment for the included studies are presented in Fig. 2.

### Quality of evidence

According to the GRADE manual, HDL-C was deemed as high-quality evidence, whereas TC, TG, LDL-C and ALT were all rated as moderate-quality evidence. FBG and AST received low-quality grades. The details of this assessment are presented in Table 2.

**Table 2:** GRADE evidence profile

Outcomes	Certainty assessment						Effect			Certainty
	①	②	③	④	⑤	⑥	No. of Studies	No. of Individuals	Rate (95%CI)	
TC	Not serious	Very serious	Not serious	Not serious	Not serious	Large effect	9RCTs	629	MD: -21.76 (-33.73 to -9.79)	⨁⨁⨁◯ Moderate
TGs	Not serious	Very serious	Not serious	Not serious	Not serious	Large effect	9RCTs	629	MD:-26.51 (-41.23 to -11.78)	⨁⨁⨁◯ Moderate
LDL-C	Not serious	Very serious	Not serious	Not serious	Not serious	Large effect	9RCTs	629	MD:-16.13 (-25.91 to -6.34)	⨁⨁⨁◯ Moderate
HDL-C	Not serious	Serious	Not serious	Not serious	Not serious	Large effect	9RCTs	629	MD:3.26 (1.28 to 5.25)	⨁⨁⨁⨁ High
FBG	Not serious	Very serious	Not serious	Not serious	Not serious	None	9RCTs	599	MD:-4.41 (-12.42 to 3.60)	⨁⨁◯◯ Low
AST	Not serious	Very serious	Not serious	Not serious	Not serious	None	9RCTs	583	MD:-4.41 (-12.42 to 3.60)	⨁⨁◯◯ Low
ALT	Not serious	Very serious	Not serious	Not serious	Not serious	Large effect	9RCTs	584	MD:-5.50 (-9.77 to -1.23)	⨁⨁⨁◯ Moderate

Notes: *TC* Total cholesterol, *TGs* Triglycerides, *LDL-C* Low-density lipoprotein cholesterol, *HDL-C* High-density lipoprotein cholesterol, *FBG* Fasting blood glucose, *AST* Aspartate aminotransferase, *ALT* Alanine aminotransferase

*CI* Confidence intervals, *MD* Mean difference

① Risk of bias

② Inconsistency

③ Directness

④ Imprecision

⑤ Publication Bias

⑥ Other considerations

### Effects of medicinal and food homologous substances on TC levels

Nine studies [18–23, 25, 28, 29] reported the effects of medicinal and food homologous substances on TC. These studies included 629 participants, 337 in the intervention group and 292 in the control group. In one study, 50 patients with metabolic fatty liver [18] were treated with Tartary buckwheat combined with sea buckthorn vinegar drink. The TC levels in the intervention group exhibited a decrease, although the difference was not statistically significant. In two studies [19, 20], hawthorn was administered for the treatment of NAFLD and demonstrated a significant effect on reducing TC levels. Ginger intervention [21] can significantly reduce TC levels; cinnamon and purslane [28, 29] have also been demonstrated to effectively reduce TC levels. One study [25] comprised three experimental groups: turmeric, chicory, and a combination of turmeric and chicory. In all three groups, a significant reduction in TC was observed. However, a separate study [23] found that the use of turmeric did not have a significant effect on TCl levels in patients with NAFLD. Similarly, the study on cardamom [22] showed no significant impact on TC levels in NAFLD patients.

### Effects of medicinal and food homologous substances on TGs levels

Nine studies [18–23, 25, 28, 29] reported the effects of medicinal and food homologous substances on TGs. These studies involved a total of 629 participants, with 337 in the intervention group and 292 in the control group. In studies of hawthorn [19, 20], sea buckthorn [18], cardamom [22], turmeric [23] and cinnamon [28], the TGs levels of patients with NAFLD were significantly reduced. In the studies involving the three experimental groups [25], the intervention of turmeric alone or turmeric combined with chicory seed could significantly reduce TGs levels. However, no significant improvement was found in the studies of ginger [21] and purslane [29].

### Effects of medicinal and food homologous substances on LDL-C levels

Nine studies [18–23, 25, 28, 29] have reported the effects of medicinal and food homologous substances, including hawthorn, sea buckthorn, ginger, cardamom, turmeric, chicory seed and purslane, on LDL-C levels; all of these substances significantly improved LDL-C levels.

### Effects of medicinal and food homologous substances on HDL-C levels

Nine studies [18–23, 25, 28, 29] reported the effects of medicinal and food homologous substances on HDL-C levels; ginger, cinnamon, and purslane [21, 28, 29] did not exhibit a significant impact on HDL-C levels. In the turmeric study [23], the turmeric group exhibited a significant improvement in HDL-C levels compared to baseline. Another study on turmeric [25] found that both the turmeric group and the combined chicory seed-turmeric group showed a significant increase in HDL-C levels. In addition, hawthorn, sea buckthorn and cardamom exhibited significant improvements in HDL-C levels.

### Effects of medicinal and food homologous substances on fasting blood glucose levels

Nine studies [18, 19, 21–23, 26–29] reported the effects of medicinal and food homologous substances, including hawthorn, sea buckthorn, ginger, cardamom, turmeric, cinnamon and puraca, on fasting blood glucose levels. Except for the study of cardamom [22], which found no significant effect on NAFLD, other researches have demonstrated a significant effect on fasting blood glucose levels.

### Effects of medicinal and food homologous substances on the levels of liver enzyme AST

Nine studies [18, 19, 21, 23–25, 27, 28, 30] reported the effects of medicinal and food homologous substances on AST. These studies included 583 participants, 317 in the intervention group and 266 in the control group. In the two studies involving ginger intervention [21, 24], there was no significant effect on the reduction of AST; in the saffron intervention studies [30], there was no significant effect on the reduction of AST. In the two turmeric studies [25, 27], no significant effect was found on the reduction of AST whether turmeric alone or chicory seed combined. However, in the study [23], turmeric played a significant role in reducing AST. Moreover, hawthorn, sea buckthorn and cinnamon [18, 19, 28] can improve AST levels in patients with NAFLD.

### Effects of medicinal and food homologous substances on the levels of liver enzyme ALT

Nine studies [18, 19, 21, 23–25, 27, 28, 30] reported the effects of medicinal and food homologous substances on alanine aminotransferase (ALT) levels. These studies included 583 participants, 317 in the intervention group and 266 in the control group. Hawthorn [19], sea buckthorn [18], ginger [21, 24], turmeric [23, 25] and cinnamon [28] all significantly reduced alanine aminotransferase levels. However, another study on turmeric [27] did not find that turmeric intervention significantly improved alanine aminotransferase levels, and saffron [30] did not significantly improve alanine aminotransferase levels.

### Summary of results

Due to the high diversity of materials included in the study, this study opted against performing a meta-analysis. The nine medicinal and food homologous substances included can improve the blood lipid levels, blood glucose levels and liver function in patients with NAFLD to a certain extent. However, the impact of turmeric on TC, AST and ALT levels remains a topic of controversy.

Furthermore, this study also summarizes the observations and conclusions included in the research, as shown in Table 3 (In the table file).

**Table 3:** The observations and conclusions of the included literature

Ref	Observations	Conclusions
[18]	After 3 months, the improvement rate of fatty liver grading in the observation group was 52.0%, which was higher than that in control group (22.9%) (*P* < 0.05)	Combined drinking of tartary buckwheat and sea buckthorn vinegar can effectively improve the fatty liver grading of MAFLD patients. It is not considered that combined drinking of Tartary buckwheat and sea buckthorn vinegar can help reduce the blood glucose, blood fat and liver function of MAFLD patients
[19]	Ninety days of Tai Chi soft ball combined with Hawthorn tea can significantly reduce the content of ALT, AST, TG, TC, LDL-C and FBG in NAFLD patients (*P* < 0.01) and increase the level of HDL-C (*P* < 0.01). This effect is better than that of the control group (*P* < 0.05)	Hawthorn tea combined with people’s recreational, economical and effective Tai Chi soft ball exercise for fat reduction, weight control and disease prevention; the benign intervention effect is very obvious
[20]	The HDL-C, LDL-C, TG and TC levels in experimental group were better than those in control group, and the difference was statistically significant (*P* < 0.05). The total clinical effective rate of the experimental group was 93. 33%, which was higher than that of the control group (76.67%), and the difference was statistically significant (*P* < 0.05)	A long-term, large dose of hawthorn can reduce blood lipid levels and improve clinical efficiency in patients with nonalcoholic fatty liver caused by high fat diet
[21]	At the end of the study, serum levels of ALT, TC, LDL-C and FBG in the group receiving a ginger supplement were significantly decreased compared to placebo	The ginger supplement may be used as a complementary therapy along with existing therapies to reduce insulin resistance, liver enzymes and inflammation in patients with nonalcoholic fatty liver
[22]	Compared with placebo, cardamom capsules significantly increased HDL-c levels and decreased TG and LDL-C levels and the grade of fatty liver (*P* < 0.05)	Cardamom capsules supplementation improved the grade of fatty liver and lipids among overweight or obese NAFLD patients
[23]	At the end of the study, the Turmeric group showed a significant reduction in liver enzymes (AST before 26.81 ± 10.54 after 21.19 ± 5.67, *P* = 0.044, ALT before 39.56 ± 22.41, after 30.51 ± 12.61, *P* = 0.043) compared with the placebo group. The serum levels of TG, LDL and HDL were significantly decreased in the turmeric group as compared to baseline, and there was no significant change in placebo group (*P* < 0.05)	This study suggests that daily consumption of turmeric (and its active phenolic ingredients as curcumin) supplementation could be effective in management of NAFLD and decreasing serum level of liver transaminases
[24]	Ginger supplementation resulted in a significant reduction in ALT, γ-glutamyl transferase, and inflammatory cytokine levels, the insulin resistance index and hepatic steatosis grade in comparison to the placebo	Twelve weeks of two grams of ginger supplementation showed beneficial effects on some NAFLD characteristics
[25]	Significant decreases in BMI and waist circumference were observed in the subjects in CHI and TUR + CHI groups, compared with those in the PLA group (*p* < 0.05). Serum levels of HDL-C were considerably increased in the TUR and TUR + CHI groups (*p* < 0.05 vs. placebo). Turmeric supplementation alone and plus chicory seed led to significant reduction in serum levels of TG/HDL-C and LDL-C/HDL-C ratio in TUR and TUR + CHI groups in comparison with the placebo (*p* < 0.05)	Turmeric and chicory seed supplementation can be useful in the management of NAFLD risk factors
[26]	Serum levels of glucose and insulin and the homeostasis model assessment for insulin resistance vales were significantly decreased in the turmeric group (by 1.22%, 17.69% and 19.48%, *P* = 0.039, *P* = 0.013 and *P* = 0.001, respectively) compared to the placebo at the end of the study	Turmeric consumption may be useful in the management of risk factors in NAFLD patients
[27]	Turmeric consumption decreased serum levels of glucose and insulin, the homeostasis model assessment of insulin resistance values, and leptin levels (by 1.22, 17.69, 19.48 and 21.33%, respectively, *p* < 0.05 for all) over 12 weeks compared with those variables in the placebo group	Turmeric supplementation improved glucose indices and serum leptin levels and may be useful in the control of NAFLD complications
[28]	In the treatment group (*P* < 0.05), significant decreases in FBG,TC,TG, ALT, AST levels were seen, but there was no significant change in serum high-density lipoproteins levels (*P* = 0 .122). In both groups, low-density lipoprotein levels decreased significantly (*P* < 0 .05)	The study suggests that taking 1500 mg cinnamon daily may be effective in improving NAFLD characteristics
[29]	Intake of purslane seeds with the low‐calorie diet led to a significant decrease in serum concentrations of FBG (− 3.52 ± 10.45 compared with 3.03 ± 9.01 mg/dl, *P* = 0.017), TC (4.33 ± 34.04 compared with 23.48 ± 29.47 mg/dl, *P* = 0.032), and LDL‐C (− 4.35 ± 22.65 compared with 11.82 ± 16.08 mg/dl, *P* = 0.004) after intervention	Compared with that in the control group, purslane seed consumption with adherence to a low‐calorie diet had beneficial effects on FBG and LDL‐C levels in patients with NAFLD but did not affect other glycemic, lipid profile, and oxidative stress parameters
[30]	In the treatment group, significant decreases in leptin (− 0.27 ng/ml, 95% CI = − 0.65, − 0.10, *p* = 0.040) and malondialdehyde (− 1.01 ng/ml, 95% CI = − 1.89, − 0.14, *p* = 0.023) levels and a significant increase in total antioxidant capacity (0.34 μmol/L, 95% CI = 0.08, 0.61, *p* = 0.011) were observed compared to the placebo group	In the present study, 12 weeks of 100 mg of saffron supplementation indicated beneficial effects on the serum levels of some inflammatory, oxidative stress, and adipokines biomarkers, but it had no significant effect on serum concentrations of liver enzymes and anthropometric and body composition measurements

## Discussion

All 13 articles included in this study were RCTs. The majority of the studies included were evaluated as having some concerns or low-risk bias. Each study compared the subjects' age, sex, education level, levels of blood lipids and glucose, liver function, and other characteristics at the beginning of the study. There was no statistically significant difference in the baseline data (*P* > 0.05).

In this study, it was found that medicinal and food homologous substances could improve blood lipid and blood glucose levels and liver function in NAFLD patients to a certain extent. Hawthorn, sea buckthorn, ginger, cinnamon, purslane and chicory seeds were able to significantly lower cholesterol. The medicinal and food homologous substances that reduced triglyceride levels were hawthorn, sea buckthorn, cardamom, cinnamon, turmeric and chicory seed. The medicinal and food homologous substances that could improve LDL-C levels were hawthorn, sea buckthorn, ginger, cardamom, turmeric, chicory seed and purslane. The medicinal and food homologous substances that increased HDL-C levels were hawthorn, sea buckthorn, cardamom, turmeric, and chicory seed. Hawthorn, sea buckthorn, ginger, turmeric, cinnamon and purslane were the medicinal and food homologous substances that significantly improved fasting blood glucose levels. Hawthorn, sea buckthorn and cinnamon were the medicinal and food homologous substances that could improve AST levels. Hawthorn, sea buckthorn, ginger and cinnamon were the medicinal and food homologous substances that could improve ALT levels, but the effect of turmeric on TC, AST and ALT levels was controversial. Although saffron does not significantly affect liver enzyme levels, the intervention has a favorable impact on serum levels of inflammation, oxidative stress, and adipokine biomarkers.

Currently, there is a growing trend of systematic evaluations being conducted on medicinal and food homologous substances both domestically and internationally [31–33]. However, they are all focused on a single substance and have not been discussed and analyzed from the category of medicinal and food homologous substances. Therefore, this systematic evaluation was carried out. In a meta-analysis examining the effects of sea-buckthorn on factors related to metabolic syndrome [31], it was determined that sea-buckthorn can effectively improve blood lipid levels in individuals with unhealthy lipid profiles, while having no significant impact on those who are already healthy. A systematic review was conducted to investigate the effects of turmeric on liver enzymes in patients with NAFLD [32], the study found that high-dose administration of turmeric can be used as a treatment for NAFLD. This treatment improved liver enzyme levels, and it is believed that it has the best effect within a specific time of supplementation. In addition, a number of animal experiments [34, 35] have also shown that medicinal and food homologous substances can improve lipid metabolism in nonalcoholic fatty liver animal models and have a protective effect on hepatic steatosis, which is consistent with the conclusion of this study. The medicinal and food homologous substances included in this study were hawthorn, sea buckthorn, purslane, turmeric, chicory seed, ginger, saffron, cardamom and cinnamon, but the number of studies on these individual substances was less than 5.

Intervention with medicinal and food homologous substances in patients with NAFLD may potentially benefit them by improving blood glucose, blood lipids, and liver function. However, the extent of these benefits may vary depending on factors such as the type of intervention substance used, timing of intervention, dosage administered, and other related variables. Among the included literature, intervention duration ranged from 8 weeks to 3 months and various types of intervention substances were utilized. In one study [25], a group of participants received joint interventions with two medicinal and food homologous substances. Furthermore, inconsistencies in dosage were observed when the same medicinal and food homologous substances were used across different studies. Moreover, NAFLD is associated with dietary and lifestyle factors. Among the studies included, three [18, 19, 29] incorporated additional interventions such as health education, dietary guidance, and tai chi exercise assistance in conjunction with medicinal and food homologous substance interventions. Ten studies [18, 21–25, 27–30] evaluated the dietary intake of study participants. These variables have the potential to impact the findings of this investigation.

In China, the use of medicinal and food homologous substances has a long history and is documented extensively in medical classics. In Iran, which is a country also interested in traditional herbal treatments, there are approximately 2,300 different types of medicinal plants with therapeutic value [36]. All studies included in the present research are from China and Iran, which may be related to the local gastronomic and medical traditions. The homologous substances of medicine and food contain rich nonnutrients, which are different from the chemical structure of nutrients. Furthermore, these substances can sustain the physiological processes of the human body and prevent several disorders. By mitigating oxidative stress and reducing inflammation, these substances have the potential to impede the progression of chronic diseases. It is worth noting that adhering to healthy dietary habits over an extended period can significantly enhance human health [37].

For the treatment of chronic diseases, this research group proposed the theoretical model of family nurse diet. In this model, the top represents nonnutrients, the bottom is an equilateral triangle made up of oxidative stress, inflammation, and metabolic problems, and the height is the dietary prescription from the family nurse. The model has been proposed to aid in the management of chronic diseases by means of its anti-inflammatory, antioxidant, and metabolic regulatory properties [37]. The lipid metabolism of patients with NAFLD is disturbed, and transaminase and blood glucose levels are higher than those of the normal population [38]. Basic research [39] found that nonalcoholic fatty liver is closely related to inflammation, oxidative stress and metabolic disorders. Quercetin, curcumin, proanthocyanidins, and other phenolic compounds are significant nonnutrients and are linked to human health. They are among the most abundant nonnutrients found in medicinal and food homologous substances. The majority of edible plants include phenolic acids and flavonoids and display immune enhancing, anti-infective, antioxidative, antiviral, and antibacterial effects [37]. In addition, the flavonoids found in many medicinal and food homologous substances have protective effects against liver damage, can control blood cholesterol levels, can act as anti-inflammatory and anticancer compounds and have a few preventive abilities against diabetes and hyperlipidemia [40, 41]. The abundant flavonoids in hawthorn may have pharmacodynamic effects on processes such as regulating cell growth and survival, lowering lipoprotein lipase levels, and minimizing inflammatory reactions [42]. The flavonoids present in seabuckthorn have the potential to reduce blood viscosity, enhance vascular compliance, augment blood circulation, lower low-density lipoprotein and cholesterol levels, as well as ameliorate serum cholesterol and insulin resistance among patients with NAFLD [43]. Purslane, which is an annual succulent herb, is also an important medicinal and food homologous plant. In animal studies, the total flavonoids extracted from purslane have been shown to mitigate oxidative stress and inflammation, as well as regulate abnormal lipid metabolism in cases of liver injury induced by NAFLD in mice [44]. Curcumin extracted from turmeric has demonstrated remarkable in vivo activities, including potent antioxidant, anti-inflammatory, antifibrotic, antiaging and antitumor effects; these properties may be responsible for the liver-protective properties of turmeric [45]. Moreover, chicory lowers blood fat levels and protects the liver. Additionally, its ingredient chlorogenic acid has an anti-obesity impact and enhances lipid metabolism [46]. The therapeutic benefits of ginger may be attributed to its ability to modulate transcription factors, particularly compounds like 6-gingerol that can regulate the expression of key genes involved in lipid metabolism and inflammation while also suppressing hepatic steatosis [47, 48]. Saffron or crocin can prevent NAFLD-mediated oxidative stress and inflammatory reactions by lowering liver enzyme levels and slowing histological alterations [35]. Cardamom has been demonstrated to possess pharmacological activities that are closely associated with human health, including antioxidant, anti-inflammatory, hypoglycemic, and hepatoprotective effects. Notably, its extract can significantly mitigate liver tissue inflammation and necrosis as well as collagen accumulation and activation of hepatic stellate cells, thereby exerting a beneficial effect on liver protection [49]. Cinnamon polyphenols, extracted from cinnamon bark, have demonstrated the ability to mitigate insulin resistance in adipocytes and liver cells while also regulating intracellular lipid metabolism [50]. This study suggests that the above medicinal and food homologs may assist disease prevention and treatment through anti-inflammation, antioxidative stress and improvement of metabolic disorders by utilizing the nonnutrients rich in them. In a randomized controlled study investigating the effects of omega-3 rich camelina sativa oil intervention in patients with NAFLD [51], it was observed that camelina sativa oil supplementation significantly improved glycemic control, inflammation and oxidative stress biomarkers in NAFLD patients. Another study [52] also demonstrated that dietary supplementation with camelina sativa oil had a positive impact on certain liver enzymes, lipid profiles, and other indicators in patients with NAFLD. This finding further supports the premise of our study. Many clinical studies have not deeply studied the active components of medicinal and food homologous substances, but their liver protection and anti-inflammatory effects make them of great developmental value.

At present, the management of chronic diseases has become the primary task of current health care. Clinical and laboratory investigations have demonstrated that the development of most chronic diseases is intricately linked to oxidative stress, inflammation, and metabolic dysregulation [53, 54], and healthy dietary patterns are effective in disease intervention, guiding the formulation and implementation of tertiary prevention strategies for chronic noncommunicable diseases at the macro level [37]. The dietary regimen based on the concept of food and medicine sharing the same origin can serve as a tertiary prevention strategy for disease prevention and health promotion in the general population, while also playing a positive role in treating and rehabilitating patients with NAFLD. This provides ideas for clinical nondrug intervention and management of the NAFLD population.

Six [21–23, 25, 26, 29] of the 13 RCTs included in the present analysis showed no negative effects after using medicinal and food homologous substances. In six studies [18–20, 24, 27, 28], no adverse effects were reported. One patient in the intervention group in one of the studies [30] was excluded due to saffron allergy, but no adverse responses were reported in the other investigations. Medicinal and food homologous substances are low-cost supplemental natural drugs that can postpone the development of NAFLD and lessen the side effects and adverse responses associated with conventional drug treatment. Medicinal and food homologous substances are safe and affordable treatment options. The nine medicinal and food homologous substances involved in this study all have certain beneficial effects for patients with NAFLD, providing a basis and guidance for clinical dietary guidance for patients with NAFLD. It is possible to develop personalized dietary adjuvant therapy for patients with NAFLD according to related indicators to prevent the development of the disease and promote the recovery of patients.

### Strengths and limitations

The strength of this study lies in the credibility of its findings, which are derived from randomized controlled trials and based on studies of overall good quality. However, this study has some limitations. Firstly, the included studies were limited to China and Iran, which may limit the generalizability of this research findings. Secondly, this research search strategy was restricted to Chinese and English articles only, potentially leading to important studies being overlooked and affecting the overall results. In addition, the technique, dosage, and timing of intervention with medicinal and food homologous substances in the included studies varied, which may have impacted the evaluation of their efficacy; therefore, meta-analysis was not conducted in this study.

## Conclusion

The results indicated that the utilization of medicinal and food homologous substances exhibited a significant improvement in serum lipid levels (TC, TGs, LDL-C, and HDL-C), FBG, as well as liver enzymes (ALT and AST) among patients diagnosed with NAFLD. However, the impact of turmeric on TC, AST, and ALT remains a topic of debate. In addition, saffron has been shown to have no significant impact on hepatic enzymes, while exhibiting favorable effects on serum levels of inflammation, oxidative stress, and adipokine biomarkers. This may be related to the intervention time and dose of medicinal and food homologous substances. Medicinal and food homologous substances have fewer side effects and are safer, economical and rich in nonnutrients compared with drugs. This study presents a novel adjuvant therapy regimen and dietary guidance for patients with NAFLD, which plays a pivotal role in the prevention and management of this disease.

### Supplementary Information

**Additional file 1.** The search strategy.

**Additional file 2.** PRISMA 2020 Checklist.

## Data Availability

The original data involved in the manuscript can be obtained from the references.

## Abbreviations

AST: Aspartate aminotransferase
ALT: Alanine aminotransferase
FBG: Fasting blood glucose
HDL-C: High-density lipoprotein cholesterol
LDL-C: Low-density lipoprotein cholesterol
NAFLD: Nonalcoholic fatty liver disease
TC: Total cholesterol
TGs: Triglycerides
