# Supplementary material for: The effects of medicinal and food homologous substances on blood lipid and blood glucose levels and liver function in patients with nonalcoholic fatty liver disease: a systematic review of randomized controlled trials
Source: Lipids Health Dis. 2023 Aug 29;22:137. doi: 10.1186/s12944-023-01900-5 (PMC10464055; doi:10.1186/s12944-023-01900-5)
Supplement: Supplementary file 1 — Additional file 1. The search strategy. [file 12944_2023_1900_MOESM1_ESM.docx]

**The effects of medicinal and food homologous substances on blood lipid and blood glucose levels and liver function in patients with nonalcoholic fatty liver disease: A systematic review of randomized controlled trials**

**The search strategy：**

**Eight databases, including Chinese National Knowledge Infrastructure(CNKI), China Science and Technology Journal Database(VIP), China Biomedical Literature** **Database(CBM), Wanfang Database, Pubmed, Cochrane Library, Embase, and Web of Science, were searched using the combination of MeSH terms and free words. The retrieval period was from the inception of the database to December 21, 2022.**

1. **In Chinese databases, the search was conducted considering the CNKI database as an example of a retrieval strategy:**

**（SU %= 'MAFLD' OR** **SU %= '非酒精性脂肪肝' OR SU %= '代谢性脂肪肝' OR SU %= '代谢相关性脂肪性肝病' OR SU %= 'NAFLD' ）AND（** **TKA = '丁香' OR TKA = '八角茴香' OR TKA= '刀豆' OR TKA= '小茴香' OR TKA = '小蓟' OR TKA = '山药' OR TKA = '山楂' OR TKA = '马齿苋' OR TKA = '乌梢蛇' OR TKA = '乌梅' OR TKA = '木瓜' OR TKA = '火麻仁' OR TKA = '代代花' OR TKA = '玉竹' OR TKA = '甘草' OR TKA= '白芷' OR TKA = '白果' OR TKA = '白扁豆' OR TKA = '桂圆' OR TKA = '决明子' OR TKA= '百合' OR TKA = '肉豆蔻' OR TKA = '肉桂' OR TKA = '余甘子' OR TKA = '佛手' OR TKA = '杏仁' OR TKA ='沙棘' OR TKA= '牡蛎' OR TKA = '芡实' OR TKA = '花椒' OR TKA= '赤小豆' OR TKA = '阿胶' OR TKA = '鸡内金' OR TKA = '麦芽' OR TKA = '昆布' OR TKA = '枣' OR TKA = '罗汉果' OR TKA = '郁李仁' OR TKA = '金银花' OR TKA = '青果' OR TKA = '鱼腥草' OR TKA = '姜' OR TKA = '枳椇子' OR TKA = '枸杞子' OR TKA = '栀子' OR TKA = '砂仁' OR TKA = '胖大海' OR TKA = '茯苓' OR TKA = '香橼' OR TKA = '香薷' OR TKA = '桃仁' OR TKA = '桑叶' OR TKA = '桑葚' OR TKA = '桔红' OR TKA = '桔梗' OR TKA = '益智仁' OR TKA = '荷叶' OR TKA = '莱菔子' OR TKA = '莲子' OR TKA = '淡竹叶' OR TKA = '淡豆豉' OR TKA = '菊花' OR TKA = '菊苣' OR TKA = '黄芥子' OR TKA = '黄精' OR TKA = '紫苏' OR TKA = '葛根' OR TKA = '黑芝麻' OR TKA = '黑胡椒' OR TKA = '槐米' OR TKA = '槐花' OR TKA = '蒲公英' OR TKA = '蜂蜜' OR TKA = '榧子' OR TKA = '酸枣仁' OR TKA = '芫荽' OR TKA = '山银花' OR TKA = '人参' OR TKA = '藿香' OR TKA = '覆盆子' OR TKA = '薤白' OR TKA = '薏苡仁' OR TKA = '薄荷' OR TKA = '橘皮' OR TKA = '蝮蛇' OR TKA = '鲜芦根' OR TKA = '鲜白茅根' OR TKA = '玫瑰花' OR TKA = '凉粉草' OR TKA = '夏枯草' OR TKA = '布渣叶' OR TKA = '鸡蛋花' OR TKA = '当归' OR TKA = '山奈' OR TKA = '西红花' OR TKA = '草果' OR TKA = '姜黄' OR TKA = '荜茇' OR TKA = '党参' OR TKA = '肉苁蓉' OR TKA = '铁皮石斛' OR TKA = '西洋参' OR TKA = '黄芪' OR TKA = '灵芝' OR TKA = '天麻' OR TKA = '山茱萸' OR TKA = '杜仲叶'）**

1. **In Chinese databases, the search was conducted considering the Wanfang database as an example of a retrieval strategy:**#1 AND #2 AND #3

#1

**[(主题:(NAFLD) or 主题:(MAFLD) or 主题:(代谢性脂肪肝) or 主题:(代谢相关性脂肪肝) or 主题:(非酒精性脂肪肝)) and (题名或关键词:(罗汉果) or 题名或关键词:(郁李仁) or 题名或关键词:(金银花) or 题名或关键词:(青果) or 题名或关键词:(鱼腥草) or 题名或关键词:(姜) or 题名或关键词:(枸杞子) or 题名或关键词:(栀子) or 题名或关键词:(枣) or 题名或关键词:(玫瑰花) or 题名或关键词:(松花粉) or 题名或关键词:(粉葛) or 题名或关键词:(布渣叶) or 题名或关键词:(夏枯草) or 题名或关键词:(当归) or 题名或关键词:(山奈) or 题名或关键词:(西红花) or 题名或关键词:(草果) or 题名或关键词:(姜黄) or 题名或关键词:(荜茇) or 题名或关键词:(党参) or 题名或关键词:(肉苁蓉) or 题名或关键词:(铁皮石斛) or 题名或关键词:(西洋参) or 题名或关键词:(黄芪) or 题名或关键词:(灵芝) or 题名或关键词:(天麻) or 题名或关键词:(山茱萸) or 题名或关键词:(杜仲叶) or 题名或关键词:(郁李仁) or 题名或关键词:(金银花) or 题名或关键词:(青果) or 题名或关键词:(砂仁) or 题名或关键词:(胖大海) or 题名或关键词:(茯苓) or 题名或关键词:(香橼) or 题名或关键词:(香薷) or 题名或关键词:(桃仁) or 题名或关键词:(桑叶))](https://s-wanfangdata-com-cn-443.webvpn.bjmu.edu.cn/advanced-search/paper?q=(%E4%B8%BB%E9%A2%98:(%E8%84%82%E8%82%AA%E8%82%9D) or %E4%B8%BB%E9%A2%98:(%E8%84%82%E8%82%AA%E6%80%A7%E8%82%9D%E7%97%85) or %E4%B8%BB%E9%A2%98:(%E4%BB%A3%E8%B0%A2%E6%80%A7%E8%84%82%E8%82%AA%E8%82%9D) or %E4%B8%BB%E9%A2%98:(%E4%BB%A3%E8%B0%A2%E7%9B%B8%E5%85%B3%E6%80%A7%E8%84%82%E8%82%AA%E8%82%9D) or %E4%B8%BB%E9%A2%98:(%E9%9D%9E%E9%85%92%E7%B2%BE%E6%80%A7%E8%84%82%E8%82%AA%E8%82%9D)) and(%E9%A2%98%E5%90%8D%E6%88%96%E5%85%B3%E9%94%AE%E8%AF%8D:(%E7%BD%97%E6%B1%89%E6%9E%9C) or %E9%A2%98%E5%90%8D%E6%88%96%E5%85%B3%E9%94%AE%E8%AF%8D:(%E9%83%81%E6%9D%8E%E4%BB%81) or %E9%A2%98%E5%90%8D%E6%88%96%E5%85%B3%E9%94%AE%E8%AF%8D:(%E9%87%91%E9%93%B6%E8%8A%B1) or %E9%A2%98%E5%90%8D%E6%88%96%E5%85%B3%E9%94%AE%E8%AF%8D:(%E9%9D%92%E6%9E%9C) or %E9%A2%98%E5%90%8D%E6%88%96%E5%85%B3%E9%94%AE%E8%AF%8D:(%E9%B1%BC%E8%85%A5%E8%8D%89) or %E9%A2%98%E5%90%8D%E6%88%96%E5%85%B3%E9%94%AE%E8%AF%8D:(%E5%A7%9C)or %E9%A2%98%E5%90%8D%E6%88%96%E5%85%B3%E9%94%AE%E8%AF%8D:(%E6%9E%B8%E6%9D%9E%E5%AD%90) or %E9%A2%98%E5%90%8D%E6%88%96%E5%85%B3%E9%94%AE%E8%AF%8D:(%E6%A0%80%E5%AD%90) or %E9%A2%98%E5%90%8D%E6%88%96%E5%85%B3%E9%94%AE%E8%AF%8D:(%E6%9E%A3) or %E9%A2%98%E5%90%8D%E6%88%96%E5%85%B3%E9%94%AE%E8%AF%8D:(%E7%8E%AB%E7%91%B0%E8%8A%B1) or %E9%A2%98%E5%90%8D%E6%88%96%E5%85%B3%E9%94%AE%E8%AF%8D:(%E6%9D%BE%E8%8A%B1%E7%B2%89) or %E9%A2%98%E5%90%8D%E6%88%96%E5%85%B3%E9%94%AE%E8%AF%8D:(%E7%B2%89%E8%91%9B) or %E9%A2%98%E5%90%8D%E6%88%96%E5%85%B3%E9%94%AE%E8%AF%8D:(%E5%B8%83%E6%B8%A3%E5%8F%B6) or %E9%A2%98%E5%90%8D%E6%88%96%E5%85%B3%E9%94%AE%E8%AF%8D:(%E5%A4%8F%E6%9E%AF%E8%8D%89) or %E9%A2%98%E5%90%8D%E6%88%96%E5%85%B3%E9%94%AE%E8%AF%8D:(%E5%BD%93%E5%BD%92) or %E9%A2%98%E5%90%8D%E6%88%96%E5%85%B3%E9%94%AE%E8%AF%8D:(%E5%B1%B1%E5%A5%88) or %E9%A2%98%E5%90%8D%E6%88%96%E5%85%B3%E9%94%AE%E8%AF%8D:(%E8%A5%BF%E7%BA%A2%E8%8A%B1) or %E9%A2%98%E5%90%8D%E6%88%96%E5%85%B3%E9%94%AE%E8%AF%8D:(%E8%8D%89%E6%9E%9C) or %E9%A2%98%E5%90%8D%E6%88%96%E5%85%B3%E9%94%AE%E8%AF%8D:(%E5%A7%9C%E9%BB%84) or %E9%A2%98%E5%90%8D%E6%88%96%E5%85%B3%E9%94%AE%E8%AF%8D:(%E8%8D%9C%E8%8C%87) or %E9%A2%98%E5%90%8D%E6%88%96%E5%85%B3%E9%94%AE%E8%AF%8D:(%E5%85%9A%E5%8F%82) or %E9%A2%98%E5%90%8D%E6%88%96%E5%85%B3%E9%94%AE%E8%AF%8D:(%E8%82%89%E8%8B%81%E8%93%89) or %E9%A2%98%E5%90%8D%E6%88%96%E5%85%B3%E9%94%AE%E8%AF%8D:(%E9%93%81%E7%9A%AE%E7%9F%B3%E6%96%9B) or %E9%A2%98%E5%90%8D%E6%88%96%E5%85%B3%E9%94%AE%E8%AF%8D:(%E8%A5%BF%E6%B4%8B%E5%8F%82) or %E9%A2%98%E5%90%8D%E6%88%96%E5%85%B3%E9%94%AE%E8%AF%8D:(%E9%BB%84%E8%8A%AA) or %E9%A2%98%E5%90%8D%E6%88%96%E5%85%B3%E9%94%AE%E8%AF%8D:(%E7%81%B5%E8%8A%9D) or %E9%A2%98%E5%90%8D%E6%88%96%E5%85%B3%E9%94%AE%E8%AF%8D:(%E5%A4%A9%E9%BA%BB) or %E9%A2%98%E5%90%8D%E6%88%96%E5%85%B3%E9%94%AE%E8%AF%8D:(%E5%B1%B1%E8%8C%B1%E8%90%B8) or %E9%A2%98%E5%90%8D%E6%88%96%E5%85%B3%E9%94%AE%E8%AF%8D:(%E6%9D%9C%E4%BB%B2%E5%8F%B6) or %E9%A2%98%E5%90%8D%E6%88%96%E5%85%B3%E9%94%AE%E8%AF%8D:(%E9%83%81%E6%9D%8E%E4%BB%81) or %E9%A2%98%E5%90%8D%E6%88%96%E5%85%B3%E9%94%AE%E8%AF%8D:(%E9%87%91%E9%93%B6%E8%8A%B1) or %E9%A2%98%E5%90%8D%E6%88%96%E5%85%B3%E9%94%AE%E8%AF%8D:(%E9%9D%92%E6%9E%9C) or %E9%A2%98%E5%90%8D%E6%88%96%E5%85%B3%E9%94%AE%E8%AF%8D:(%E7%A0%82%E4%BB%81) or %E9%A2%98%E5%90%8D%E6%88%96%E5%85%B3%E9%94%AE%E8%AF%8D:(%E8%83%96%E5%A4%A7%E6%B5%B7) or %E9%A2%98%E5%90%8D%E6%88%96%E5%85%B3%E9%94%AE%E8%AF%8D:(%E8%8C%AF%E8%8B%93) or %E9%A2%98%E5%90%8D%E6%88%96%E5%85%B3%E9%94%AE%E8%AF%8D:(%E9%A6%99%E6%A9%BC) or %E9%A2%98%E5%90%8D%E6%88%96%E5%85%B3%E9%94%AE%E8%AF%8D:(%E9%A6%99%E8%96%B7) or %E9%A2%98%E5%90%8D%E6%88%96%E5%85%B3%E9%94%AE%E8%AF%8D:(%E6%A1%83%E4%BB%81) or %E9%A2%98%E5%90%8D%E6%88%96%E5%85%B3%E9%94%AE%E8%AF%8D:(%E6%A1%91%E5%8F%B6))&type=["periodical","thesis","conference"]&chineseEnglishExpand=true" \t "https://s-wanfangdata-com-cn-443.webvpn.bjmu.edu.cn/advanced-search/_blank)**

#2

**[(主题:(NAFLD) or 主题:(MAFLD) or 主题:(代谢性脂肪肝) or 主题:(代谢相关性脂肪肝) or 主题:(非酒精性脂肪肝)) and (题名或关键词:(桑葚) or 题名或关键词:(桔红) or 题名或关键词:(桔梗) or 题名或关键词:(益智仁) or 题名或关键词:(荷叶) or 题名或关键词:(莱菔子) or 题名或关键词:(莲子) or 题名或关键词:(高良姜) or 题名或关键词:(淡竹叶) or 题名或关键词:(淡豆豉) or 题名或关键词:(菊花) or 题名或关键词:(菊苣) or 题名或关键词:(黄芥子) or 题名或关键词:(紫苏) or 题名或关键词:(紫苏籽) or 题名或关键词:(葛根) or 题名或关键词:(黑芝麻) or 题名或关键词:(黑胡椒) or 题名或关键词:(槐米) or 题名或关键词:(槐花) or 题名或关键词:(蒲公英) or 题名或关键词:(蜂蜜) or 题名或关键词:(榧子) or 题名或关键词:(酸枣仁) or 题名或关键词:(鲜白茅根) or 题名或关键词:(鲜芦根) or 题名或关键词:(蝮蛇) or 题名或关键词:(橘皮) or 题名或关键词:(薄荷) or 题名或关键词:(薏苡仁) or 题名或关键词:(薤白) or 题名或关键词:(覆盆子) or 题名或关键词:(藿香) or 题名或关键词:(人参) or 题名或关键词:(山银花) or 题名或关键词:(芫荽))](https://s-wanfangdata-com-cn-443.webvpn.bjmu.edu.cn/advanced-search/paper?q=(%E4%B8%BB%E9%A2%98:(%E8%84%82%E8%82%AA%E8%82%9D) or %E4%B8%BB%E9%A2%98:(%E8%84%82%E8%82%AA%E6%80%A7%E8%82%9D%E7%97%85) or %E4%B8%BB%E9%A2%98:(%E4%BB%A3%E8%B0%A2%E6%80%A7%E8%84%82%E8%82%AA%E8%82%9D) or %E4%B8%BB%E9%A2%98:(%E4%BB%A3%E8%B0%A2%E7%9B%B8%E5%85%B3%E6%80%A7%E8%84%82%E8%82%AA%E8%82%9D) or %E4%B8%BB%E9%A2%98:(%E9%9D%9E%E9%85%92%E7%B2%BE%E6%80%A7%E8%84%82%E8%82%AA%E8%82%9D)) and(%E9%A2%98%E5%90%8D%E6%88%96%E5%85%B3%E9%94%AE%E8%AF%8D:(%E6%A1%91%E8%91%9A) or %E9%A2%98%E5%90%8D%E6%88%96%E5%85%B3%E9%94%AE%E8%AF%8D:(%E6%A1%94%E7%BA%A2) or %E9%A2%98%E5%90%8D%E6%88%96%E5%85%B3%E9%94%AE%E8%AF%8D:(%E6%A1%94%E6%A2%97) or %E9%A2%98%E5%90%8D%E6%88%96%E5%85%B3%E9%94%AE%E8%AF%8D:(%E7%9B%8A%E6%99%BA%E4%BB%81) or %E9%A2%98%E5%90%8D%E6%88%96%E5%85%B3%E9%94%AE%E8%AF%8D:(%E8%8D%B7%E5%8F%B6) or %E9%A2%98%E5%90%8D%E6%88%96%E5%85%B3%E9%94%AE%E8%AF%8D:(%E8%8E%B1%E8%8F%94%E5%AD%90) or %E9%A2%98%E5%90%8D%E6%88%96%E5%85%B3%E9%94%AE%E8%AF%8D:(%E8%8E%B2%E5%AD%90) or %E9%A2%98%E5%90%8D%E6%88%96%E5%85%B3%E9%94%AE%E8%AF%8D:(%E9%AB%98%E8%89%AF%E5%A7%9C) or %E9%A2%98%E5%90%8D%E6%88%96%E5%85%B3%E9%94%AE%E8%AF%8D:(%E6%B7%A1%E7%AB%B9%E5%8F%B6) or %E9%A2%98%E5%90%8D%E6%88%96%E5%85%B3%E9%94%AE%E8%AF%8D:(%E6%B7%A1%E8%B1%86%E8%B1%89) or %E9%A2%98%E5%90%8D%E6%88%96%E5%85%B3%E9%94%AE%E8%AF%8D:(%E8%8F%8A%E8%8A%B1) or %E9%A2%98%E5%90%8D%E6%88%96%E5%85%B3%E9%94%AE%E8%AF%8D:(%E8%8F%8A%E8%8B%A3) or %E9%A2%98%E5%90%8D%E6%88%96%E5%85%B3%E9%94%AE%E8%AF%8D:(%E9%BB%84%E8%8A%A5%E5%AD%90) or %E9%A2%98%E5%90%8D%E6%88%96%E5%85%B3%E9%94%AE%E8%AF%8D:(%E7%B4%AB%E8%8B%8F) or %E9%A2%98%E5%90%8D%E6%88%96%E5%85%B3%E9%94%AE%E8%AF%8D:(%E7%B4%AB%E8%8B%8F%E7%B1%BD) or %E9%A2%98%E5%90%8D%E6%88%96%E5%85%B3%E9%94%AE%E8%AF%8D:(%E8%91%9B%E6%A0%B9) or %E9%A2%98%E5%90%8D%E6%88%96%E5%85%B3%E9%94%AE%E8%AF%8D:(%E9%BB%91%E8%8A%9D%E9%BA%BB) or %E9%A2%98%E5%90%8D%E6%88%96%E5%85%B3%E9%94%AE%E8%AF%8D:(%E9%BB%91%E8%83%A1%E6%A4%92) or %E9%A2%98%E5%90%8D%E6%88%96%E5%85%B3%E9%94%AE%E8%AF%8D:(%E6%A7%90%E7%B1%B3) or %E9%A2%98%E5%90%8D%E6%88%96%E5%85%B3%E9%94%AE%E8%AF%8D:(%E6%A7%90%E8%8A%B1) or %E9%A2%98%E5%90%8D%E6%88%96%E5%85%B3%E9%94%AE%E8%AF%8D:(%E8%92%B2%E5%85%AC%E8%8B%B1) or %E9%A2%98%E5%90%8D%E6%88%96%E5%85%B3%E9%94%AE%E8%AF%8D:(%E8%9C%82%E8%9C%9C) or %E9%A2%98%E5%90%8D%E6%88%96%E5%85%B3%E9%94%AE%E8%AF%8D:(%E6%A6%A7%E5%AD%90) or %E9%A2%98%E5%90%8D%E6%88%96%E5%85%B3%E9%94%AE%E8%AF%8D:(%E9%85%B8%E6%9E%A3%E4%BB%81) or %E9%A2%98%E5%90%8D%E6%88%96%E5%85%B3%E9%94%AE%E8%AF%8D:(%E9%B2%9C%E7%99%BD%E8%8C%85%E6%A0%B9) or %E9%A2%98%E5%90%8D%E6%88%96%E5%85%B3%E9%94%AE%E8%AF%8D:(%E9%B2%9C%E8%8A%A6%E6%A0%B9) or %E9%A2%98%E5%90%8D%E6%88%96%E5%85%B3%E9%94%AE%E8%AF%8D:(%E8%9D%AE%E8%9B%87) or %E9%A2%98%E5%90%8D%E6%88%96%E5%85%B3%E9%94%AE%E8%AF%8D:(%E6%A9%98%E7%9A%AE) or %E9%A2%98%E5%90%8D%E6%88%96%E5%85%B3%E9%94%AE%E8%AF%8D:(%E8%96%84%E8%8D%B7) or %E9%A2%98%E5%90%8D%E6%88%96%E5%85%B3%E9%94%AE%E8%AF%8D:(%E8%96%8F%E8%8B%A1%E4%BB%81) or %E9%A2%98%E5%90%8D%E6%88%96%E5%85%B3%E9%94%AE%E8%AF%8D:(%E8%96%A4%E7%99%BD) or %E9%A2%98%E5%90%8D%E6%88%96%E5%85%B3%E9%94%AE%E8%AF%8D:(%E8%A6%86%E7%9B%86%E5%AD%90) or %E9%A2%98%E5%90%8D%E6%88%96%E5%85%B3%E9%94%AE%E8%AF%8D:(%E8%97%BF%E9%A6%99) or %E9%A2%98%E5%90%8D%E6%88%96%E5%85%B3%E9%94%AE%E8%AF%8D:(%E4%BA%BA%E5%8F%82) or %E9%A2%98%E5%90%8D%E6%88%96%E5%85%B3%E9%94%AE%E8%AF%8D:(%E5%B1%B1%E9%93%B6%E8%8A%B1) or %E9%A2%98%E5%90%8D%E6%88%96%E5%85%B3%E9%94%AE%E8%AF%8D:(%E8%8A%AB%E8%8D%BD))&type=["periodical","thesis","conference"]&chineseEnglishExpand=true" \t "https://s-wanfangdata-com-cn-443.webvpn.bjmu.edu.cn/advanced-search/_blank)**

#3

**([主题:(NAFLD) or 主题:(MAFLD) or 主题:(代谢性脂肪肝) or 主题:(代谢相关性脂肪肝) or 主题:(非酒精性脂肪肝)) and （题名或关键词:(八角茴香) or 题名或关键词:(小茴香) or 题名或关键词:(山药) or 题名或关键词:(马齿苋) or 题名或关键词:(乌梅) or 题名或关键词:(火麻仁) or 题名或关键词:(玉竹) or 题名或关键词:(白芷) or 题名或关键词:(白扁豆) or 题名或关键词:(桂圆) or 题名或关键词:(百合) or 题名或关键词:(肉桂) or 题名或关键词:(佛手) or 题名或关键词:(沙棘) or 题名或关键词:(芡实) or 题名或关键词:(阿胶) or 题名或关键词:(麦芽) or 题名或关键词:(枣) or 题名或关键词:(罗汉果) or 题名或关键词:(金银花) or 题名或关键词:(鱼腥草) or 题名或关键词:(枳椇子) or 题名或关键词:(枸杞子) or 题名或关键词:(砂仁) or 题名或关键词:(茯苓) or 题名或关键词:(香薷) or 题名或关键词:(桑叶) or 题名或关键词:(桔红) or 题名或关键词:(益智仁) or 题名或关键词:(莱菔子） or 题名或关键词:(高良姜) or 题名或关键词:(淡豆豉) or 题名或关键词:(菊苣) or 题名或关键词:(黄精) or 题名或关键词:(紫苏) or 题名或关键词:(黑芝麻) or 题名或关键词:(槐米) or 题名或关键词:(蒲公英) or 题名或关键词:(酸枣仁) or 题名或关键词:(橘皮) or 题名或关键词:(黄芪) or 题名或关键词:(党参) )](https://s-wanfangdata-com-cn-443.webvpn.bjmu.edu.cn/advanced-search/paper?q=(%E4%B8%BB%E9%A2%98:(%E8%84%82%E8%82%AA%E8%82%9D) or %E4%B8%BB%E9%A2%98:(%E8%84%82%E8%82%AA%E6%80%A7%E8%82%9D%E7%97%85) or %E4%B8%BB%E9%A2%98:(%E4%BB%A3%E8%B0%A2%E6%80%A7%E8%84%82%E8%82%AA%E8%82%9D) or %E4%B8%BB%E9%A2%98:(%E4%BB%A3%E8%B0%A2%E7%9B%B8%E5%85%B3%E6%80%A7%E8%84%82%E8%82%AA%E8%82%9D) or %E4%B8%BB%E9%A2%98:(%E9%9D%9E%E9%85%92%E7%B2%BE%E6%80%A7%E8%84%82%E8%82%AA%E8%82%9D)) and %EF%BC%88%E9%A2%98%E5%90%8D%E6%88%96%E5%85%B3%E9%94%AE%E8%AF%8D:(%E5%85%AB%E8%A7%92%E8%8C%B4%E9%A6%99) or %E9%A2%98%E5%90%8D%E6%88%96%E5%85%B3%E9%94%AE%E8%AF%8D:(%E5%B0%8F%E8%8C%B4%E9%A6%99) or %E9%A2%98%E5%90%8D%E6%88%96%E5%85%B3%E9%94%AE%E8%AF%8D:(%E5%B1%B1%E8%8D%AF) or %E9%A2%98%E5%90%8D%E6%88%96%E5%85%B3%E9%94%AE%E8%AF%8D:(%E9%A9%AC%E9%BD%BF%E8%8B%8B) or %E9%A2%98%E5%90%8D%E6%88%96%E5%85%B3%E9%94%AE%E8%AF%8D:(%E4%B9%8C%E6%A2%85) or %E9%A2%98%E5%90%8D%E6%88%96%E5%85%B3%E9%94%AE%E8%AF%8D:(%E7%81%AB%E9%BA%BB%E4%BB%81) or %E9%A2%98%E5%90%8D%E6%88%96%E5%85%B3%E9%94%AE%E8%AF%8D:(%E7%8E%89%E7%AB%B9) or %E9%A2%98%E5%90%8D%E6%88%96%E5%85%B3%E9%94%AE%E8%AF%8D:(%E7%99%BD%E8%8A%B7) or %E9%A2%98%E5%90%8D%E6%88%96%E5%85%B3%E9%94%AE%E8%AF%8D:(%E7%99%BD%E6%89%81%E8%B1%86) or %E9%A2%98%E5%90%8D%E6%88%96%E5%85%B3%E9%94%AE%E8%AF%8D:(%E6%A1%82%E5%9C%86) or %E9%A2%98%E5%90%8D%E6%88%96%E5%85%B3%E9%94%AE%E8%AF%8D:(%E7%99%BE%E5%90%88) or %E9%A2%98%E5%90%8D%E6%88%96%E5%85%B3%E9%94%AE%E8%AF%8D:(%E8%82%89%E6%A1%82) or %E9%A2%98%E5%90%8D%E6%88%96%E5%85%B3%E9%94%AE%E8%AF%8D:(%E4%BD%9B%E6%89%8B) or %E9%A2%98%E5%90%8D%E6%88%96%E5%85%B3%E9%94%AE%E8%AF%8D:(%E6%B2%99%E6%A3%98) or %E9%A2%98%E5%90%8D%E6%88%96%E5%85%B3%E9%94%AE%E8%AF%8D:(%E8%8A%A1%E5%AE%9E) or %E9%A2%98%E5%90%8D%E6%88%96%E5%85%B3%E9%94%AE%E8%AF%8D:(%E9%98%BF%E8%83%B6) or %E9%A2%98%E5%90%8D%E6%88%96%E5%85%B3%E9%94%AE%E8%AF%8D:(%E9%BA%A6%E8%8A%BD) or %E9%A2%98%E5%90%8D%E6%88%96%E5%85%B3%E9%94%AE%E8%AF%8D:(%E6%9E%A3) or %E9%A2%98%E5%90%8D%E6%88%96%E5%85%B3%E9%94%AE%E8%AF%8D:(%E7%BD%97%E6%B1%89%E6%9E%9C) or %E9%A2%98%E5%90%8D%E6%88%96%E5%85%B3%E9%94%AE%E8%AF%8D:(%E9%87%91%E9%93%B6%E8%8A%B1) or %E9%A2%98%E5%90%8D%E6%88%96%E5%85%B3%E9%94%AE%E8%AF%8D:(%E9%B1%BC%E8%85%A5%E8%8D%89) or %E9%A2%98%E5%90%8D%E6%88%96%E5%85%B3%E9%94%AE%E8%AF%8D:(%E6%9E%B3%E6%A4%87%E5%AD%90) or %E9%A2%98%E5%90%8D%E6%88%96%E5%85%B3%E9%94%AE%E8%AF%8D:(%E6%9E%B8%E6%9D%9E%E5%AD%90) or %E9%A2%98%E5%90%8D%E6%88%96%E5%85%B3%E9%94%AE%E8%AF%8D:(%E7%A0%82%E4%BB%81) or %E9%A2%98%E5%90%8D%E6%88%96%E5%85%B3%E9%94%AE%E8%AF%8D:(%E8%8C%AF%E8%8B%93) or %E9%A2%98%E5%90%8D%E6%88%96%E5%85%B3%E9%94%AE%E8%AF%8D:(%E9%A6%99%E8%96%B7) or %E9%A2%98%E5%90%8D%E6%88%96%E5%85%B3%E9%94%AE%E8%AF%8D:(%E6%A1%91%E5%8F%B6) or %E9%A2%98%E5%90%8D%E6%88%96%E5%85%B3%E9%94%AE%E8%AF%8D:(%E6%A1%94%E7%BA%A2) or %E9%A2%98%E5%90%8D%E6%88%96%E5%85%B3%E9%94%AE%E8%AF%8D:(%E7%9B%8A%E6%99%BA%E4%BB%81) or %E9%A2%98%E5%90%8D%E6%88%96%E5%85%B3%E9%94%AE%E8%AF%8D:(%E8%8E%B1%E8%8F%94%E5%AD%90%EF%BC%89 or %E9%A2%98%E5%90%8D%E6%88%96%E5%85%B3%E9%94%AE%E8%AF%8D:(%E9%AB%98%E8%89%AF%E5%A7%9C) or %E9%A2%98%E5%90%8D%E6%88%96%E5%85%B3%E9%94%AE%E8%AF%8D:(%E6%B7%A1%E8%B1%86%E8%B1%89) or %E9%A2%98%E5%90%8D%E6%88%96%E5%85%B3%E9%94%AE%E8%AF%8D:(%E8%8F%8A%E8%8B%A3) or %E9%A2%98%E5%90%8D%E6%88%96%E5%85%B3%E9%94%AE%E8%AF%8D:(%E9%BB%84%E7%B2%BE) or %E9%A2%98%E5%90%8D%E6%88%96%E5%85%B3%E9%94%AE%E8%AF%8D:(%E7%B4%AB%E8%8B%8F) or %E9%A2%98%E5%90%8D%E6%88%96%E5%85%B3%E9%94%AE%E8%AF%8D:(%E9%BB%91%E8%8A%9D%E9%BA%BB) or %E9%A2%98%E5%90%8D%E6%88%96%E5%85%B3%E9%94%AE%E8%AF%8D:(%E6%A7%90%E7%B1%B3) or %E9%A2%98%E5%90%8D%E6%88%96%E5%85%B3%E9%94%AE%E8%AF%8D:(%E8%92%B2%E5%85%AC%E8%8B%B1) or %E9%A2%98%E5%90%8D%E6%88%96%E5%85%B3%E9%94%AE%E8%AF%8D:(%E9%85%B8%E6%9E%A3%E4%BB%81) or %E9%A2%98%E5%90%8D%E6%88%96%E5%85%B3%E9%94%AE%E8%AF%8D:(%E6%A9%98%E7%9A%AE) or %E9%A2%98%E5%90%8D%E6%88%96%E5%85%B3%E9%94%AE%E8%AF%8D:(%E9%BB%84%E8%8A%AA) or %E9%A2%98%E5%90%8D%E6%88%96%E5%85%B3%E9%94%AE%E8%AF%8D:(%E5%85%9A%E5%8F%82) )&type=["periodical","thesis","conference"]&chineseEnglishExpand=true" \t "https://s-wanfangdata-com-cn-443.webvpn.bjmu.edu.cn/advanced-search/_blank)**

1. **In Chinese databases, the search was conducted considering the VIP database as an example of a retrieval strategy:**#1 AND #2

#1

**(****非酒精性脂肪肝 OR 代谢性脂肪肝 OR 代谢相关性脂肪性肝病 OR NAFLD OR MAFLD) AND （****丁香 OR 八角茴香 OR 刀豆 OR 小茴香 OR 小蓟 OR 山药 OR 山楂 OR 马齿苋 OR 乌梢蛇 OR 乌梅 OR 木瓜 OR 火麻仁 OR 代代花 OR 玉竹 OR 甘草 OR 白芷 OR 白果 OR 白扁豆 OR 桂圆 OR 决明子 OR 百合 OR 肉豆蔻 OR 肉桂 OR 余甘子 OR 佛手 OR 杏仁 OR 沙棘 OR 牡蛎 OR 芡实 OR 花椒 OR 赤小豆 OR 阿胶 OR 鸡内金 OR 麦芽 OR 昆布 OR 枣 OR 罗汉果 OR 郁李仁 OR 金银花 OR 青果 OR 鱼腥草 OR 姜 OR 枳椇子 OR 枸杞子 OR 栀子 OR 砂仁 OR 胖大海 OR 茯苓 OR 香橼 OR 香薷 OR 桃仁 OR 桑叶 OR 桑葚 OR 桔红 OR 桔梗 OR 益智仁 OR 荷叶 OR 莱菔子 OR 莲子 OR 淡竹叶 OR 淡豆豉)**

#2

**(非酒精性脂肪肝 OR 代谢性脂肪肝 OR 代谢相关性脂肪性肝病 OR NAFLD OR MAFLD) AND OR 菊花 OR 菊苣 OR 黄芥子 OR 黄精 OR 紫苏 OR 葛根 OR 黑芝麻 OR 黑胡椒 OR 槐米 OR 槐花 OR 蒲公英 OR 蜂蜜 OR 榧子 OR 酸枣仁 OR 芫荽 OR 山银花 OR 人参 OR 藿香 OR 覆盆子 OR 薤白 OR 薏苡仁 OR 薄荷 OR 橘皮 OR 蝮蛇 OR 鲜芦根 OR 鲜白茅根 OR 玫瑰花 OR 凉粉草 OR 夏枯草 OR 布渣叶 OR 鸡蛋花 OR 当归 OR 山奈 OR 西红花 OR 草果 OR 姜黄 OR 荜茇 OR 党参 OR 肉苁蓉 OR 铁皮石斛 OR 西洋参 OR 黄芪 OR 灵芝 OR 天麻 OR 山茱萸 OR 杜仲叶）**

1. **In Chinese databases, the search was conducted considering the CBM database as an example of a retrieval strategy:**

**(非酒精性脂肪肝 OR 代谢性脂肪肝 OR 代谢相关性脂肪性肝病 OR NAFLD OR MAFLD) AND（丁香 OR 八角茴香 OR 刀豆 OR 小茴香 OR 小蓟 OR 山药 OR 山楂 OR 马齿苋 OR 乌梢蛇 OR 乌梅 OR 木瓜 OR 火麻仁 OR 代代花 OR 玉竹 OR 甘草 OR 白芷 OR 白果 OR 白扁豆 OR 桂圆 OR 决明子 OR 百合 OR 肉豆蔻 OR 肉桂 OR 余甘子 OR 佛手 OR 杏仁 OR 沙棘 OR 牡蛎 OR 芡实 OR 花椒 OR 赤小豆 OR 阿胶 OR 鸡内金 OR 麦芽 OR 昆布 OR 枣 OR 罗汉果 OR 郁李仁 OR 金银花 OR 青果 OR 鱼腥草 OR 姜 OR 枳椇子 OR 枸杞子 OR 栀子 OR 砂仁 OR 胖大海 OR 茯苓 OR 香橼 OR 香薷 OR 桃仁 OR 桑叶 OR 桑葚 OR 桔红 OR 桔梗 OR 益智仁 OR 荷叶 OR 莱菔子 OR 莲子 OR 淡竹叶 OR 淡豆豉 OR 菊花 OR 菊苣 OR 黄芥子 OR 黄精 OR 紫苏 OR 葛根 OR 黑芝麻 OR 黑胡椒 OR 槐米 OR 槐花 OR 蒲公英 OR 蜂蜜 OR 榧子 OR 酸枣仁 OR 芫荽 OR 山银花 OR 人参 OR 藿香 OR 覆盆子 OR 薤白 OR 薏苡仁 OR 薄荷 OR 橘皮 OR 蝮蛇 OR 鲜芦根 OR 鲜白茅根 OR 玫瑰花 OR 凉粉草 OR 夏枯草 OR 布渣叶 OR 鸡蛋花 OR 当归 OR 山奈 OR 西红花 OR 草果 OR 姜黄 OR 荜茇 OR 党参 OR 肉苁蓉 OR 铁皮石斛 OR 西洋参 OR 黄芪 OR 灵芝 OR 天麻 OR 山茱萸 OR 杜仲叶）**

1. **For English databases, Pubmed was used as an example:**

**((((((((Non alcoholic Fatty Liver Disease[Title/Abstract]) OR (NAFLD[Title/Abstract])) OR (Nonalcoholic Fatty Liver Disease[Title/Abstract])) OR (Fatty Liver, Nonalcoholic[Title/Abstract])) OR (Metabolic associated fatty liver disease[Title/Abstract])) OR (MAFLD))) OR ("Non-alcoholic Fatty Liver Disease"[Mesh])) AND ((((((Crataegus laevigata[Title/Abstract]) OR (Crataegus monogyna[Title/Abstract])OR (Hawthorn[Title/Abstract])) OR (Hawthorns[Title/Abstract])) OR (((((((((sea-buckthorn[Title/Abstract]) ) OR (Lycium chinense[Title/Abstract])) OR (Lycium barbarum[Title/Abstract])) OR (Goji Berry[Title/Abstract])) OR (Berry Plant, Goji[Title/Abstract])) OR (Wolfberry[Title/Abstract])) OR (Goji Berry[Title/Abstract])) OR (Plants, Goji Berry[Title/Abstract]))) OR ((((((((((((((((((((((((((((((((((((((((eucommia ulmoides leaf[Title/Abstract]) OR (cornus[Title/Abstract])) OR (gastrodia[Title/Abstract])) OR (lucid ganoderma[Title/Abstract])) OR (Astragalus [Title/Abstract])) OR (American ginseng[Title/Abstract])) OR (Dendrobium officinale[Title/Abstract])) OR (cistanche[Title/Abstract])) OR (Codonopsis pilosula[Title/Abstract])) OR (piperis longi[Title/Abstract])) OR (turmeric[Title/Abstract])) OR (Amomum OR (Amomum tsao-ko[Title/Abstract])) OR (Saffron[Title/Abstract])) OR (rhizoma kaempferiae[Title/Abstract])) OR (Angelica sinensis[Title/Abstract])) OR (Agastache rugosus[Title/Abstract])) OR (fructus rubi[Title/Abstract])) OR (Longstamen Onion Bulb[Title/Abstract])) OR (coix seed[Title/Abstract])) OR (mint[Title/Abstract])) OR (orange peel[Title/Abstract])) OR (Agkistrodon halys[Title/Abstract])) OR (Fresh rehmannia root[Title/Abstract])) OR (ziziphi[Title/Abstract])) OR (semen torreya[Title/Abstract])) OR (honey[Title/Abstract])) OR (Taraxacum[Title/Abstract])) OR (Flos Sophorae[Title/Abstract])) OR (sophora flower bud[Title/Abstract])) OR (black sesame[Title/Abstract])) OR (Pueraria[Title/Abstract])) OR (perila seeds[Title/Abstract])) OR (Polygonatum[Title/Abstract])) OR (Yellow mustard[Title/Abstract])) OR (witloof[Title/Abstract])) OR (chrysanthemum[Title/Abstract])) OR (Sojae Praeparatum[Title/Abstract])) OR (lophatherum gracile[Title/Abstract])) OR (galangal[Title/Abstract])) OR (lotus seed[Title/Abstract])))) OR ((((((((((((((((((((((((((((((((((((((((((((((((((((((((clove[Title/Abstract]) OR (star anise[Title/Abstract])) OR (sword bean[Title/Abstract])) OR (cumin[Title/Abstract])) OR (Herba Cirsii[Title/Abstract])) OR (Dioscorea[Title/Abstract])) OR (Crataegus[Title/Abstract])) OR (Crataegus[Title/Abstract])) OR (Zaocys Dhumnade[Title/Abstract])) OR (dark plum[Title/Abstract])) OR (pawpaw[Title/Abstract])) OR (Hemp Seed[Title/Abstract])) OR (seville orange flower[Title/Abstract])) OR (odoratum[Title/Abstract])) OR (glycyrrhiza[Title/Abstract])) OR (radix angelicae[Title/Abstract])) OR (ginkgo[Title/Abstract])) OR (white hyacinth Bean[Title/Abstract])) OR (White lentil flowers[Title/Abstract])) OR (arillus longan[Title/Abstract])) OR (semen cassiae[Title/Abstract])) OR (lily[Title/Abstract])) OR (myristica fragrans[Title/Abstract])) OR (cinnamon[Title/Abstract])) OR (Phyllanthus emblica[Title/Abstract])) OR (fingered citron[Title/Abstract])) OR (almond[Title/Abstract])) OR (Hippophae[Title/Abstract])) OR (Semen Euryales[Title/Abstract])) OR (Sichuan Pepper[Title/Abstract])) OR (phaseolus calcaratus[Title/Abstract])) OR (colla corii asini[Title/Abstract])) OR (endothelium corneum gigeriae galli[Title/Abstract])) OR (malt[Title/Abstract])) OR (sea-tangle[Title/Abstract])) OR (jujube[Title/Abstract])) OR (Siraitia grosvenorii[Title/Abstract])) OR (bunge cherry seed[Title/Abstract])) OR (honeysuckle[Title/Abstract])) OR (Chinese olive[Title/Abstract])) OR (cordate houttuynia[Title/Abstract])) OR (ginger[Title/Abstract])) OR (fructus lycii[Title/Abstract])) OR (Cape jasmine[Title/Abstract])) OR (fructus amomi[Title/Abstract])) OR (boat-fruited sterculia seed[Title/Abstract])) OR (boat-fruited sterculia seed[Title/Abstract])) OR (citron[Title/Abstract])) OR (Chinese mosla Herb[Title/Abstract])) OR (peach seed[Title/Abstract])) OR (Mulberry Leaf[Title/Abstract])) OR (mulberry[Title/Abstract])) OR (Platycodon grandiflorum[Title/Abstract])) OR (fructus Alpiniae oxyphyllae[Title/Abstract])) OR (lotus leaf[Title/Abstract])) OR (semen raphani[Title/Abstract])))**

1. **For English databases, Embase was used as an example:** #1 AND #2

#1 ：

**'clove':ab,ti OR** **'star anise****':ab,ti OR 'sword bean****':ab,ti OR 'cumin':ab,ti OR 'herba cirsii':ab,ti OR dioscorea:ab,ti OR crataegus:ab,ti OR purslane:ab,ti OR 'zaocys dhumnade':ab,ti OR 'dark plum':ab,ti OR pawpaw:ab,ti OR 'hemp seed':ab,ti OR 'seville orange flower':ab,ti OR odoratum:ab,ti OR glycyrrhiza:ab,ti OR 'radix angelicae':ab,ti OR ginkgo:ab,ti OR 'white hyacinth bean':ab,ti OR 'white lentil flower':ab,ti OR 'arillus longan':ab,ti OR 'semen cassiae':ab,ti OR lily:ab,ti OR 'myristica fragrans':ab,ti OR cinnamon:ab,ti OR 'phyllanthus emblica':ab,ti OR 'fingered citron':ab,ti OR almond:ab,ti OR hippophae:ab,ti OR 'semen euryales':ab,ti OR 'sichuan pepper':ab,ti OR 'phaseolus calcaratus':ab,ti OR 'colla corii asini':ab,ti OR 'endothelium corneum gigeriae galli':ab,ti OR malt:ab,ti OR 'sea tent':ab,ti OR 'sea buckthorn':ab,ti OR jujube:ab,ti OR 'siraidia grosvenorii':ab,ti OR 'bunge cherry seed':ab,ti OR honeysuckle:ab,ti OR 'chinese olive':ab,ti OR 'cordate houttuynia':ab,ti OR ginger:ab,ti OR 'japanese raisintree fruit':ab,ti OR 'fructus lycii':ab,ti OR 'cape jasmine':ab,ti OR 'fructus amomi':ab,ti OR 'boat-fruited sterculia seed':ab,ti OR wolfiporia:ab,ti OR citron:ab,ti OR 'chinese mosla herb':ab,ti OR 'eucommia ulmoides leaf' OR (('eucommia'/exp OR eucommia) AND ulmoides AND ('leaf'/exp OR leaf)) OR cornus:ti,ab,kw OR gastrodia:ti,ab,kw OR 'lucid ganoderma':ab,ti OR astragalus:ab,ti OR 'american ginseng':ab,ti OR 'dendrobium officinale':ab,ti OR cistanche:ab,ti OR 'codonopsis pilosula':ab,ti OR 'piperis longi':ab,ti OR turmeric:ab,ti OR 'amomum tsao-ko':ab,ti OR saffron:ab,ti OR 'rhizoma kaempferiae':ab,ti OR 'angelica sinensis':ab,ti OR 'agastache rugosus':ab,ti OR 'fructus rubi':ab,ti OR 'longstamen onion bulb':ab,ti OR 'coix seed':ab,ti OR mint:ab,ti OR 'orange peel':ab,ti OR 'agkistrodon halys':ab,ti OR 'fresh rehmannia root':ab,ti OR 'fresh rhizoma imperatae':ab,ti OR 'ziziphi spinosae':ab,ti OR 'semen torreya':ab,ti OR honey:ab,ti OR taraxacum:ab,ti OR 'flos sophorae':ab,ti OR 'sophora flower bud':ab,ti OR 'black sesame':ab,ti OR pueraria:ab,ti OR 'perila seeds':ab,ti OR kingianum:ab,ti OR 'yellow mustard':ab,ti OR witloof:ab,ti OR chrysanthemum:ab,ti OR praeparatum:ab,ti OR 'lophatherum gracile':ab,ti OR galangal:ab,ti OR 'lotus seed':ab,ti OR 'semen raphani':ab,ti OR 'lotus leaf':ab,ti OR oxyphyllae:ab,ti OR platycodon:ab,ti OR mulberry:ab,ti OR 'mulberry leaf':ab,ti OR 'peach seed':ab,ti OR crataegus:ab,ti OR 'crataegus laevigata':ab,ti OR 'crataegus monogyna':ab,ti OR hawthorn:ab,ti OR hawthorns:ab,ti OR lycium:ab,ti OR 'lycium chinense':ab,ti OR 'goji berry plant':ab,ti OR 'berry plant, goji':ab,ti OR 'plant, goji berry':ab,ti**

#2：

**'nonalcoholic fatty liver'/exp OR 'non alcoholic fatty liver disease':ab,ti OR nafld:ab,ti OR 'nonalcoholic fatty liver disease':ab,ti OR 'fatty liver, nonalcoholic':ab,ti OR 'metabolic associated fatty liver disease':ab,ti OR 'non-alcoholic fatty liver':ab,ti OR mafld:ab,ti**

1. **For English databases, Web of science was used as an example:** #1 AND #2

#1

TS=Non alcoholic Fatty Liver Disease OR TS=NAFLD OR TS=Nonalcoholic Fatty Liver Disease OR TS=Fatty Liver, Nonalcoholic OR TS=Metabolic associated fatty liver disease OR TS=Non-alcoholic Fatty Liver Disease

#2

**TS=clove OR TS=star anise OR TS=sword bean OR TS=cumin OR TS=Herba Cirsii OR TS=Dioscorea OR TS=Crataegus OR TS=purslane OR TS=Zaocys Dhumnade OR TS=dark plum OR TS=pawpaw OR TS=Hemp Seed OR TS=seville orange flower OR TS=odoratum OR TS=glycyrrhiza OR TS=radix angelicae OR TS=ginkgo OR TS=white hyacinth Bean OR TS=White lentil flower OR TS=arillus longan OR TS=semen cassiae OR TS=lily OR TS=myristica fragrans OR TS=cinnamon OR TS=Phyllanthus emblica OR TS=fingered citron OR TS=almond OR TS=Hippophae OR TS=Semen Euryales OR TS=Sichuan Pepper OR TS=phaseolus calcaratus OR TS=colla corii asini OR TS=endothelium corneum gigeriae galli OR TS=malt OR TS=sea-buckthorn OR TS=jujube OR TS=Siraidia Grosvenorii OR TS=bunge cherry seed OR TS=honeysuckle OR TS=Chinese olive OR TS=cordate houttuynia OR TS=ginger OR TS=japanese raisintree fruit OR TS=fructus lycii OR TS=Cape jasmine OR TS=fructus amomi OR TS=boat-fruited sterculia seed OR TS=Wolfiporia OR TS=citron OR TS=Chinese mosla Herb OR TS=peach seed OR TS=Mulberry Leaf OR TS=mulberry OR TS=Platycodon grandiflorum OR TS=fructus Alpiniae oxyphyllae OR TS=lotus leaf OR TS=semen raphani OR TS=lotus seed OR TS=galangal OR TS=lophatherum gracile OR TS=Semen Sojae Praeparatum OR TS=clove OR TS=star anise OR TS=sword bean OR TS=cumin OR TS=Herba Cirsii OR TS=Dioscorea OR TS=Crataegus OR TS=purslane OR TS=Zaocys Dhumnade OR TS=dark plum OR TS=pawpaw OR TS=Hemp Seed OR TS=seville orange flower OR TS=odoratum OR TS=glycyrrhiza OR TS=radix angelicae OR TS=ginkgo OR TS=white hyacinth Bean OR TS=White lentil flower OR TS=arillus longan OR TS=semen cassiae OR TS=lily OR TS=myristica fragrans OR TS=cinnamon OR TS=Phyllanthus emblica OR TS=fingered citron OR TS=almond OR TS=Hippophae OR TS=Semen Euryales OR TS=Sichuan Pepper OR TS=phaseolus calcaratus OR TS=colla corii asini OR TS=endothelium corneum gigeriae galli OR TS=malt OR TS=sea-buckthorn OR TS=jujube OR TS=Siraidia Grosvenorii OR TS=bunge cherry seed OR TS=honeysuckle OR TS=Chinese olive OR TS=cordate houttuynia OR TS=ginger OR TS=japanese raisintree fruit OR TS=fructus lycii OR TS=Cape jasmine OR TS=fructus amomi OR TS=boat-fruited sterculia seed OR TS=Wolfiporia OR TS=citron OR TS=Chinese mosla Herb OR TS=peach seed OR TS=Mulberry Leaf OR TS=mulberry OR TS=Platycodon grandiflorum OR TS=fructus Alpiniae oxyphyllae OR TS=lotus leaf OR TS=semen raphani OR TS=lotus seed OR TS=galangal OR TS=lophatherum gracile OR TS=Semen Sojae Praeparatum**

1. **For English databases, The Cochrane library was used as an example:** #1 AND #2

#1

**((clove):ti,ab,kw OR (star anise):ti,ab,kw OR (sword bean):ti,ab,kw OR (cumin):ti,ab,kw OR (Herba Cirsii):ti,ab,kw OR (Dioscorea):ti,ab,kw OR (Crataegus):ti,ab,kw OR (purslane):ti,ab,kw OR (Zaocys Dhumnade):ti,ab,kw OR (dark plum):ti,ab,kw OR (pawpaw):ti,ab,kw OR (Hemp Seed):ti,ab,kw OR (seville orange flower):ti,ab,kw OR (odoratum):ti,ab,kw OR (glycyrrhiza):ti,ab,kw OR (radix angelicae):ti,ab,kw OR (ginkgo):ti,ab,kw OR (white hyacinth Bean):ti,ab,kw OR (White lentil flowers):ti,ab,kw OR (arillus longan):ti,ab,kw OR (semen cassiae):ti,ab,kw OR (lily):ti,ab,kw OR (myristica fragrans):ti,ab,kw OR (cinnamon):ti,ab,kw OR (Phyllanthus emblica):ti,ab,kw OR (fingered citron):ti,ab,kw OR (almond):ti,ab,kw OR (Hippophae):ti,ab,kw OR (Semen Euryales):ti,ab,kw OR (Sichuan Pepper):ti,ab,kw OR (phaseolus calcaratus):ti,ab,kw OR (colla corii asini):ti,ab,kw OR (endothelium corneum gigeriae galli):ti,ab,kw OR (malt):ti,ab,kw OR (sea-buckthorn):ti,ab,kw OR (jujube):ti,ab,kw OR (Siraidia Grosvenorii):ti,ab,kw OR (bunge cherry seed):ti,ab,kw OR (honeysuckle):ti,ab,kw OR (Chinese olive):ti,ab,kw OR (cordate houttuynia):ti,ab,kw OR (ginger):ti,ab,kw OR (fructus lycii):ti,ab,kw OR (Cape jasmine):ti,ab,kw OR (fructus amomi):ti,ab,kw OR (boat-fruited sterculia seed):ti,ab,kw OR (Wolfiporia):ti,ab,kw OR (citron):ti,ab,kw OR (Chinese mosla Herb):ti,ab,kw OR (peach seed):ti,ab,kw OR (Mulberry Leaf):ti,ab,kw OR (mulberry):ti,ab,kw OR (Platycodon grandiflorum):ti,ab,kw OR (eucommia ulmoides leaf):ti,ab,kw OR (cornus):ti,ab,kw OR (gastrodia):ti,ab,kw OR (lucid ganoderma):ti,ab,kw OR (Astragalus):ti,ab,kw OR (American ginseng):ti,ab,kw OR (Dendrobium officinale):ti,ab,kw OR (gastrodia):ti,ab,kw OR (cistanche):ti,ab,kw OR (Codonopsis pilosula):ti,ab,kw OR (piperis longi):ti,ab,kw OR (turmeric):ti,ab,kw OR (Amomum tsao-ko):ti,ab,kw OR (Saffron):ti,ab,kw OR (rhizoma kaempferiae):ti,ab,kw OR (Angelica sinensis):ti,ab,kw OR (Agastache rugosus):ti,ab,kw OR (fructus rubi):ti,ab,kw OR (Longstamen Onion Bulb):ti,ab,kw OR (coix seed):ti,ab,kw OR (mint):ti,ab,kw OR (orange peel):ti,ab,kw OR (Agkistrodon halys):ti,ab,kw OR (Fresh rehmannia root):ti,ab,kw OR (Fresh rhizoma imperatae):ti,ab,kw OR (ziziphi spinosae):ti,ab,kw OR (semen torreya):ti,ab,kw OR (honey):ti,ab,kw OR (Taraxacum):ti,ab,kw OR (Flos Sophorae):ti,ab,kw OR (sophora flower bud):ti,ab,kw OR (lack sesame):ti,ab,kw OR (Pueraria):ti,ab,kw OR (perila seeds):ti,ab,kw OR (Kingianum):ti,ab,kw OR (Yellow mustard):ti,ab,kw OR (witloof):ti,ab,kw OR (chrysanthemum):ti,ab,kw OR (Praeparatum):ti,ab,kw OR (lophatherum gracile):ti,ab,kw OR (galangal):ti,ab,kw OR (lotus seed):ti,ab,kw OR (semen raphani):ti,ab,kw OR (lotus leaf):ti,ab,kw OR (Alpiniae):ti,ab,kw OR (Crataegus laevigata):ti,ab,kw OR (Crataegus monogyna):ti,ab,kw OR (Hawthorn):ti,ab,kw OR (Hawthorns):ti,ab,kw OR (Lycium chinense):ti,ab,kw OR (Lycium barbarum):ti,ab,kw OR (Goji Berry Plant):ti,ab,kw OR (Berry Plant, Goji):ti,ab,kw OR (Goji Berry):ti,ab,kw)**

#2

**(Non-alcoholic Fatty Liver Disease):ti,ab,kw OR (NAFLD):ti,ab,kw OR (Nonalcoholic Fatty Liver Disease):ti,ab,kw OR (Fatty Liver, Nonalcoholic):ti,ab,kw OR (Metabolic (dysfunction) associated fatty liver disease):ti,ab,kw**
